# A novel synbiotic delays Alzheimer’s disease onset via combinatorial gut-brain-axis signaling in *Drosophila melanogaster*

**DOI:** 10.1371/journal.pone.0214985

**Published:** 2019-04-22

**Authors:** Susan Westfall, Nikita Lomis, Satya Prakash

**Affiliations:** Biomedical and Cell Therapy Research Laboratory, Department of Biomedical Engineering and Artificial Cells and organs Research Center, Faculty of Medicine, McGill University, Montreal, Canada; Lancaster University, UNITED KINGDOM

## Abstract

The gut-brain-axis (GBA) describing the bidirectional communication between the gut microbiota and brain was recently implicated in Alzheimer’s disease (AD). The current study describes a novel synbiotic containing three metabolically active probiotics and a novel polyphenol-rich prebiotic which has beneficial impacts on the onset and progression of AD. In a transgenic humanized *Drosophila melanogaster* model of AD, the synbiotic increased survivability and motility and rescued amyloid beta deposition and acetylcholinesterase activity. Such drastic effects were due to the synbiotic’s combinatorial action on GBA signaling pathways including metabolic stability, immune signaling, oxidative and mitochondrial stress possibly through pathways implicating PPARγ. Overall, this study shows that the therapeutic potential of GBA signaling is best harnessed in a synbiotic that simultaneously targets multiple risk factors of AD.

## Introduction

The gut microbiota is a dynamic endocrine organ containing trillions of symbiotic microbes that together shape host health [1]. The bacterial community has significant interindividual variations due to lifestyle and determines some of the differences in human biochemistry and disease resistance including disorders involving the brain [2]. The gut microbiota communicates with the brain via several endocrine, neurological and biochemical pathways through a bidirectional communication system known as the gut-brain-axis (GBA) [3]. Recently, direct links between the composition of the gut microbiota with Alzheimer’s disease (AD) development have been made leaving an intriguing opportunity for the development of gut microbiota modifying products for the prevention and/or treatment of AD.

In a preclinical study, it was shown that germ-free mice overexpressing both amyloid peptide protein(APP) and presenilin (PS)1 have reduced amyloid beta (Aβ)1-42 load and microglial activation compared to conventional mice, which was reversed upon colonization with fecal matter from APP/PS1 mutants but not wild-type mice [4]. Similarly, chronic treatment with an antibiotic cocktail to the same model reduced microglial and astrocyte accumulation surrounding Aβ plaques in the hippocampus [5]. Several bacterial species have been found to produce or aggravate the production of Aβ plaques possibly through mechanisms of molecular mimicry [6] while there have been reports of increased proportions of infiltrated gram-negative bacteria and LPS in AD patients coupled with mucosal disruption in response to this dysbiosis [7]. Overall, these recent reports suggest that the gut microbiota plays a pinnacle role in the onset and progression of AD.

Supporting this, only 5% of AD cases have a familial origin and the remaining cases are attributed to environmental stochastic stresses such as inflammation, oxidative stress, insulin resistance and mitochondrial dysfunction [8], all which are influenced by the composition of the gut microbiota. Many of these risk factors are common to healthy aging individuals; however, in the case of AD patients, these risk factors are cumulative and exaggerated which together aggravate the neurological phenotype. Indeed, the authors previously observed that wildtype aging *Drosophila* have mild deficits in managing metabolic stress, inflammation and oxidative stress, and treatment with probiotic and synbiotic formulations elicited beneficial effects towards promoting longevity through coordinated activity on each of these risk factors [9]. This demonstrates that most environmental stresses can be managed by the gut microbiota and likewise, a structured regime of probiotics and prebiotics may be used to impact GBA signaling and prevent the onset and progression of AD. Indeed, several studies have demonstrated the efficacy of probiotics in the treatment of various neurological conditions [9]. For example, *Lactobacillus fermentum* NCIMB 5221 potently produces ferulic acid (FA), a phytochemical that reduces Aβ fibril formulation, neuroinflammation and restores learning and memory deficits in AD animal models [10]. The well-characterized probiotic formulation VSL#3 was shown to attenuate the age-related deficits in long-term potentiation, decreased microglial activation and downregulated several genes involved in neurodegeneration [11]. There have also been ample indirect studies implicating the gut microbiota in managing risk factors to the development of neurological diseases including insulin resistance, metabolic stress, oxidative stress, inflammation and age-related cognitive decline [9, 12]; however, never has a single gut microbiota-modulating product (probiotic, synbiotic or otherwise) been show to simultaneously influence all of these stochastic risk factors while showing preclinical benefit on AD markers.

The present study utilizes a humanized transgenic model of *Drosophila*, which is a verified model of AD. Although *Drosophila* have a native version of APP (known as APP-like or APPL) with some functional redundancy [13], APPL lacks the amyloidogenic Aβ sequence at the C terminus thus cannot be processed by the *Drosophila’s* innate γ-secretases into the fibrillogenic Aβ1–42. *Drosophila* also do not have a BACE1 homolog; however, it has been shown that overexpression of human APP can generate the Aβ peptide in *Drosophila* suggesting that another BACE-like enzyme may exist [14].

Using this model, the present study demonstrates how a synbiotic formulation containing three bioactive probiotics, *Lactobacillus plantarum* NCIMB 8826 (Lp8826), *L*. *fermentum* NCIMB 5221 (Lf5221) and *Bifidobacteria longum* spp. *infantis* NCIMB 702255 (Bi702255) and a polyphenol rich polyphenol plant extract from the gastrointestinal tonic Triphala (TFLA) [15], can simultaneously impact several aspects of GBA signaling to prevent AD onset and delay its progression possibly through mechanisms implication peroxisome proliferator activated receptor (PPAR)γ. Although both the probiotic formulation and TFLA can individually invoke benefits on some of the risk factors of AD, only the synbiotic formulation can consistently ameliorate all of the tested risk factors making the combinatorial therapy more robust than any of its constituents.

## Materials and methods

### Drosophila husbandry

*Drosophila melanogaster* were reared on a standard cornmeal-sucrose-yeast media without active yeast culture prepared by boiling the cornmeal (83 g), sucrose (50 g) and yeast extract (30 g) in distilled water for 30 min. *Drosophila* were kept in controlled conditions with a 12h:12h light-dark cycle at 25 °C. Male and female flies were separated 3–5 days following eclosion and only male flies were used for all experimental analyses. Media bottles, both treated and untreated, were changed every 3–4 days to prevent the build-up of wastes or mould. All experimental protocols were conducted with 5 independent samples of *Drosophila* using 10–25 flies per sample depending on the downstream assay.

### Probiotic cultivation

The three probiotic strains, *Lactobacillus plantarum* NCIMB 8826 (Lp8826), *Lactobacillus fermentum* NCIMB 5221 (Lf5221) and *Bifidobacteria longum* spp. *infantis* NCIMB 702255 (Bi702255) were obtained from NCIMB culture collection (Aberdeen, Scotland, UK). Cells were cultured with Man-Rogosa-Sharpe (MRS) media obtained from Sigma Aldrich (Oakville, ON, Canada) at 37 °C on MRS-agar plates or in liquid media. After one round of liquid culture, several bacterial stocks were made in MRS containing 20% (*v/v*) glycerol and stored at -80 °C. As constant culturing was required to carry out all experiments, bacterial stocks were renewed from the frozen stock bi-weekly in order to maintain culture purity. To preform each individual experiment, a 1% (*v/v*) inoculum was used for subculturing, incubated at 37 °C for 18 h and removed immediately before use.

The probiotic formulation contained a total of 3.0 x 10^9^ CFU/ml of probiotics with equal distribution between Lp8826 (1.0x10^9^ CFU/ml), Lf5221 (1.0x10^9^ CFU/ml) and Bi702255 (1.0x10^9^ CFU/ml). The synbiotic formulation contained the described probiotic formulation in combination with 0.5% of TFLA powder. The dried components of Triphala (TFLA; *Emblica officinalis*, *Terminalia bellirica* and *Terminalia chebula*) were obtained from the Ayurvedic Pharmacy at Banaras Hindu University in Varanasi, India. Each component was individually weighed and combined in equal parts (by weight) before being manually crushed and ground with a mortar and pestle. To prepare the synbiotic, 5 g of TFLA powder was combined with 1 L of the *Drosophila* media during the boiling process to make a final concentration of 0.5% TFLA in the complete media.

### Drosophila genetic model of Alzheimer’s disease

AD was modeled by the UAS-(β-secretase) BACE1-APP gene (BDSC_33797, Indiana University, Bloomington, IN) which expresses human BACE1 and the 695 amino acid isoform of human APP under the control of UAS. The UAS-BACE1-APP gene will be driven by the *elav*-GAL4 driver (BDSC_33805, Indiana University, Bloomington, IN). Expression of human BACE1 produces Aβ-like particles instigating AD pathogenesis in 3–4 weeks as described in previous studies [16].

### Physiological tests

Total lifespan and motility was assessed in *Drosophila* afflicted with AD to determine their overall fitness with age. Lifespan was calculated by first separating 10 flies into isolated vials and tallying daily the number of flies alive following their respective treatment. Each of the treatment groups were compared to control APP-BACE1 AD *Drosophila* to assess for the effect of treatment. To compare survivability between wild-type and AD *Drosophila*, the median survival in days was assessed for both the *elav*-Gal4 driver and the APP-BACE1 *Drosophila*. Motility was assessed using the negative geotaxis test. Briefly, 10 flies were lightly anesthetized with ether and transferred to an empty vial 15 cm in height. After 45 min of acclimatization, flies were gently tapped to the bottom of the vial and the time it took for 50% of the flies to reach a 10 cm mark on the vial was recorded.

### Acetylcholine esterase activity assay

ACh activity was assessed in fly head homogenates using a modified Ellman’s method [17]. Twenty fly heads were homogenized in 200 μL of ice-cold Tris-EDTA-Triton X-100 in the presence of protease inhibitors followed by centrifugation at 10,000 rpm for 2 min to remove body fragments. Following, 5 μL of homogenate was added to 100 μL of 10 mM acetylthiocholine and incubated at 37°C for 10 min to run the enzymatic reaction. The reaction was stopped with 50 μL of 6 mM Dithiobis(2-nitrobenzoic acid). After diluting with another 50 μL of buffer solution, absorbance was read at 410 nm and total ACh activity amounts was normalized to total sample protein.

### Total amyloid content

Total amyloid content was assessed using the Thioflavin T (Tht) assay protocol [18]. Similar as the ACh activity assay, 20 fly heads were homogenized in a Tris-EDTA-Triton X-100 buffer with protease inhibitors and centrifuged to remove cellular debris. A stock solution of Tht was made by diluting 8 mg of Tht into 10 mL of PBS, pH 7.0. For the working solution, the Tht stock solution was diluted 1:5 into PBS and 2 μL of sample homogenate was added to 198 μL of working Tht solution. Fluorescence was measured at 440 nm excitation / 482 nm emission and the total quantification of amyloids was normalized to total sample protein.

### Metabolic marker determination: Weight, glucose, triglycerides

Body weight was assessed by weighing 10 flies in replicates of five at the time of anesthetization. Glucose measurements were taken from both hemolymph and whole-body homogenates of *Drosophila* representing the levels of circulating and total glucose, respectively. Hemolymph was extracted by piercing anesthetized *Drosophila* with a fine tungsten needle, placing them in a small tube perforated with several holes situated in a larger tube and centrifuged for 10 min at 4000 rpm. For the whole-body homogenates, the total protein content was first determined using a Bradford Assay and resultant quantification of metabolic markers was standardized against the total protein content in order to account for variations in fly mass. Following, the homogenate was heat-treated for 20 min at 70 °C to remove any complexes. Glucose levels were measured in 2 μl of hemolymph or 5 μl of whole-body homogenate using the Glucose (HK) Assay kit (Sigma, Oakvilla, ON, Canada) according to the manufacturer’s instructions. Whole-body triglycerides were determined in 10 μl of homogenate using the Triglycerides Liquicolor Test Mono (Stanbio, TX, USA) according to the manufacture’s instructions.

### Genetic determination of metabolic and inflammatory markers

RNA was extracted from whole flies using Trizol (ThermoFisher, MA, USA) according to the manufacturer’s instructions. cDNA was synthesized from 1 μg of RNA measured with the ND-2000 Nanodrop (FisherScientific, Ottawa, ON, Canada) using the High-Capacity cDNA Synthesis Kit (ThermoFisher, MA, USA) according to the manufacturer’s instructions. Expression of various metabolic genes was assessed by real-time PCR with primers as described (S1 Table) and primers used for inflammatory markers are outlined (S2 Table). SybrGreen was used for detection with each reaction being carried out in triplicate and target gene expression compared to the housekeeping gene ribosomal protein (Rp)49 using the 2^-ddCT^ method for relative expression.

### Oxidative stress markers

Total oxidants were assayed using 2’-7’-dichlorofluorescein diacetate (DCFA) (Sigma, Oakville, ON) as previously outlined [19]. Briefly, 20 μl of fly homogenate was mixed with 170 μL of Locke’s buffer. Following, 10 μl of 1 mM DCFA solution was added to each well and after 3 min incubation and fluorescently read at 474 nm excitation and 530 nm emission wavelengths. Quantification was normalized to the amount of protein in each sample.

SOD activity was tested using a xanthine-xanthine oxidase reaction to generate superoxide radicals and nitrotetrazolium blue (NBT) reduction as an indicator of superoxide production [20]. Briefly, the working solution consisted of 110 μl of potassium phosphate buffer with 20 mg/ml BSA, 6.25 μl catalase (40 U/ml), 6.25 μl of NBT and 50 μl of xanthine (1.8 mM). To the working solution, 7.5 μl of sample and 20 μl of xanthine oxidase (XOD, 5 U/ml) was added and incubated at 37 °C for 20 min in the dark with agitation to allow colour to develop. Absorbance of reduced NBT was recorded at 570 nm, compared to a standard curve of SOD and normalized to the amount of protein in the sample.

GPx activity was assessed based on the oxidation of glutathione (GSSG), which was constantly supplied by an excess of glutathione reductase (GR). To measure GPx activity the consequent reduction of the cosubstrate NADPH was monitored at 340 nm as previous shown [21] with modifications. Briefly, a GPx buffer was made containing 0.5 M sodium phosphate (pH 7.2), 100 mM EDTA and 1.1 mM sodium azide. The GPx assay buffer consisted of 1.33 mM of GSSH and 1.33 U/ml GR in GPx buffer. The assay solution consisted of 160 μl of GPx assay solution, 10 μl of NADPH (5 mM), 20 μl hydrogen peroxide (0.5%) and 15 μl of homogenate. The absorbance at 340 nm was monitored for 3 min and the linear portion of the curve was assessed for GPx activity and quantification was normalized to the amount of protein in the sample.

The level of lipid peroxidation was assessed as bound malondialdehyde (MDA) was hydrolyzed in the presence of butylated hydroxytolene (BHT) as adapted from [22]. The reaction solution contained 10 mM of 1-metyl-2-phenylindole in a 3:1 mixture of acetonitrile:methanol. To 120 μl of this solution, 20 μl of sample and 40 μl of 37% HCl was added and allowed to react at 100 °C for 60 min. The absorbance of the resulting solution was measured at 550 nm, compared to MDA standard solution and normalized to the amount of total protein in the sample.

### Mitochondrial electron transport chain (ETC) complex activity

Mitochondria was isolated from *Drosophila* by homogenizing 40 anesthetized flies in 4 mL of ice-cold hypotonic buffer (25 mM K_2_HPO_4_ and 5 mM MgCl_2_) with a pre-cooled smooth pestle tissue grinder (Corning, NY, USA) on ice. Large pieces were filtered out using a fine nylon mesh and the remaining isolate was flash-frozen on dry ice before being stored at -80 °C. Before use, the homogenates underwent 3 freeze-thaw cycles to loosen the mitochondrial membrane.

ETC complex I (NADH–ubiquinone oxidoreductase) activity was assessed by measuring the electron transfer to decyluniquinone from NADH as previously described [23]. The assay buffer contained potassium phosphate buffer (50 mM, pH 7.2) in addition to BSA (50 mg/mL), potassium cyanide (KCN; 10 mM), NADH (10 mM) and rotenone (1 mM). To start the reaction, 25 μL of the homogenate was added to 150 μL of the assay buffer in addition to decyluniquinone (10 μM). The change in absorbance at 340 nm was monitored over two minutes and the slope of NADH reduction was recorded and normalized to the total protein in the sample.

ETC complex II (Succinate-ubiquinone oxidoreductase) activity was measured as the rate of 2,6-dicholorophenolindophenol (DCPIP) reduction as previously described [23]. The assay buffer contained potassium phosphate buffer (50 mM, pH 7.6) in addition to BSA (50 mg/mL), potassium cyanide (KCN, 10 mM), succinate (400 mM) and DCPIP (0.015% *w*/*v*). To start the reaction, 10 μL of the homogenate in addition to phenazine methosulfate (65 mM) was added to 160 μL of the assay buffer. The rate of reduction was measured at 600 nm over two minutes and the activity of ETC complex II was measured as the linear portion of the slope of DCPIP reduction and normalized to the amount of protein in the sample.

ETC complex III (NADH-cytochrome c oxidoreductase) activity was determined as the rate of antimycin dependent reduction of cytochrome c as previously described [23]. The assay buffer contained potassium phosphate buffer (50 mM, pH 7.2) in addition to oxidized cytochrome c (1mM), KCN (10 mM), EDTA (5 mM), Tween-20 (2.5%) and antimycin A (1 mg/mL). To start the reaction, 20 μL of sample homogenate was added to the assay buffer in addition to 10 μL of decyubiquiol (10 mM). The reduction of cytochrome c was monitored at 550 nm for 2 min both with and without antimycin A and the difference in the reaction rates was taken as the antimycin A sensitive complex activity and normalized to the amount of protein in the sample.

Finally, ETC complex IV (cytochrome c oxidase) activity was determined by monitoring the rate of cytochrome c oxidation [23]. The assay buffer contained potassium phosphate buffer (50 mM, pH 8.0) supplemented with reduced cytochrome c (1 mM). Cytochrome c was reduced by adding a few grains of sodium dithionite to the oxidized cytochrome c solution until the red colour turned to orange and an absorbance ratio greater of 550 nm to 565 nm was greater than 6. The reaction was initialized with the addition of 25 μl of the homogenate and the rate of reduction recorded for 3 min at 550 nm. The complex rate was normalized to the total amount of protein in the sample.

### Statistical analyses

All experiments were conducted with 5 independent trials and statistical assessments were conducted with Graphpad Prism version 7.0. Survivability data was scored using the log-rank test compared to untreated APP-BACE1 controls with Mann-Whitney tests performed for between-group comparisons of the medians. All between group data for physiological and genetic assessments were assessed using 2-way ANOVA analyses with Tukey post-hoc analyses. Significance was determined if *p* < 0.05 and evaluation at *p* < 0.01.

## Results

**Fig 1:**
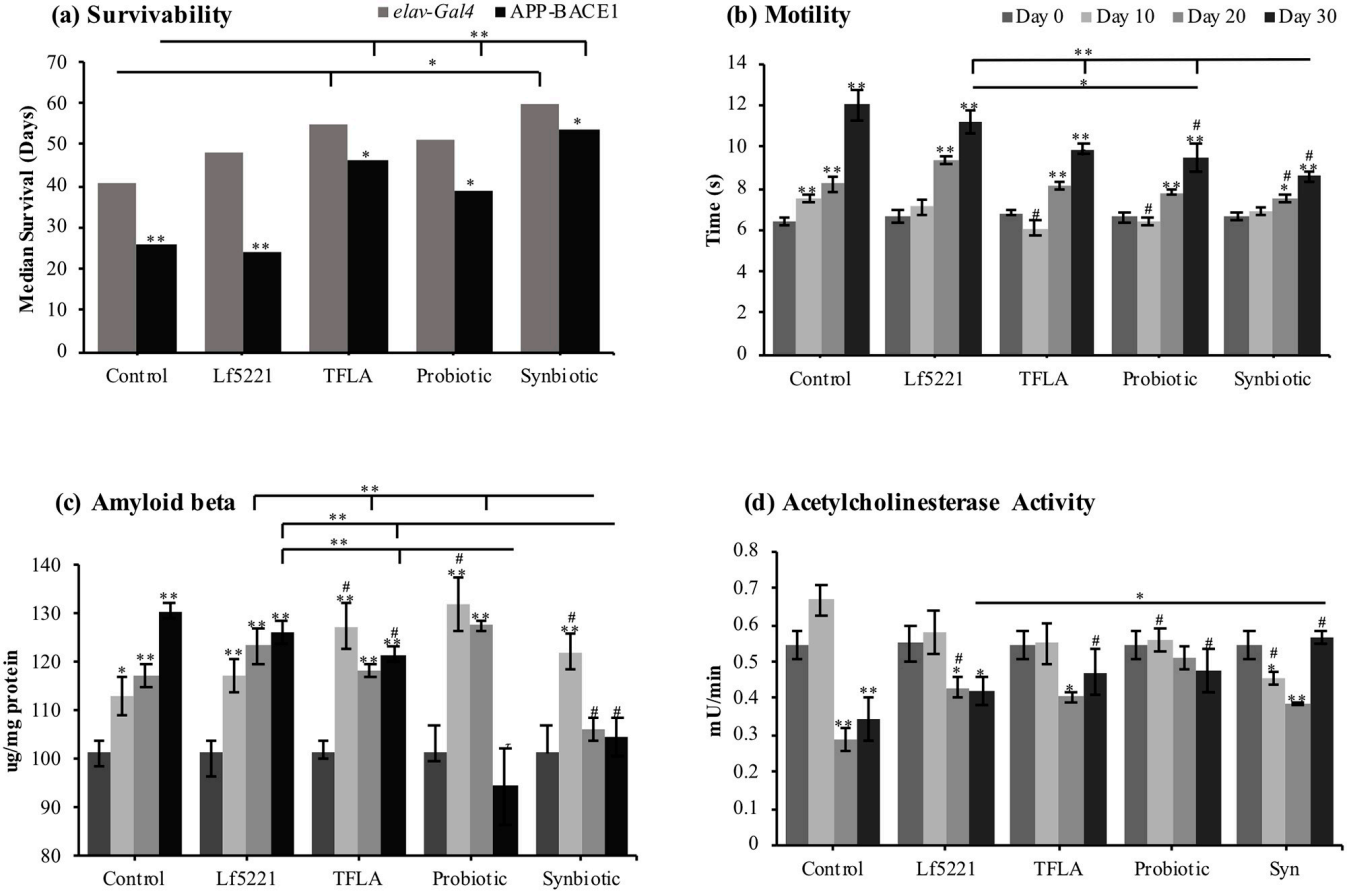
A synbiotic ameliorates markers of Alzheimer’s disease in aging *Drosophila melanogaster*. After eclosion, AD transgenic *Drosophila* (APP-BACE1) were placed on control media, or media supplemented with either Lf5221, TFLA, the probiotic or synbiotic formulation. Measures of the *Drosophila*’s (a) survivability, (b) motility, (c) accumulation of amyloid beta (Aβ) and (d) acetylcholinesterase (ACh) activity were taken at days 0, 10, 20 and 30. Each group in b, c and d is the average of n = 5 independent groups +/- SEM. Significance is indicated as black stars (*) relative to the day 0 control * p < 0.05 and ** p < 0.01 or tau (#) relative to the non-supplemented control at the same time point where # implies p < 0.05. The lifespan analysis is the result of a single lifespan analysis where significant between groups in one genotype is measured with the log-rank test and between-group median of the *elav*-Gal4 and APP-BACE1 group through Mann-Whitney tests.

To test the impact of the probiotic, prebiotic and their combination on the survivability of both wildtype and AD *Drosophila*, 5 independent groups of 10 male *Drosophila* were isolated after eclosion from each treatment group either with the *elav*-Gal4 only genetic background (wildtype) or the crossed APP-BACE1 AD model. Untreated control AD *Drosophila* showed 80% survivability at day 15 and 40% at day 30. At day 15, survivability was improved by all treatment groups with the synbiotic group retaining a 100% survivability. By day 30, the Lf5221 group had the lowest survivability at 30%, the TFLA and probiotic groups at 60% and the synbiotic group was significantly higher at 75% survivability (S1 Fig). Using the log-rank test in the AD model compared to the untreated APP-BACE1 control, no significant variations in lifespan were observed in the Lf5221 group (*p*>0.05) while the TFLA (*p*<0.05), probiotic (*p*<0.05) and synbiotic (*p*<0.05) groups all significantly improved survivability (S1 Fig). To control for the impact of normal aging, the median survivability of the wildtype *elav*-Gal4 driver line was compared to the APP-BACE1 AD model. In the non-treated control group, the median survivability of the *elav-Gal4* group was significantly higher than the control APP-BACE1 group, as expected. Further, there was no difference between the *elav-Gal4* control and APP-BACE1 lines following TFLA or synbiotic treatment. In addition, the probiotic treatment significantly improved survivability of the APP-BACE1 group compared to the no-treatment control whereas in the *elav-Gal4* group, the probiotic treatment elicited no such effect. Together, this suggests that the impact of the TFLA, probiotic or synbiotic interventions is more pronounced in the APP-BACE1 AD group compared to the control *elav-Gal4* aging group suggesting that the differences in survivability is not only reflecting normal aging (S1 Fig). Comparing survivability of the *elav*-Gal4 flies with the APP-BACE1 flies both treated with either Lf5221, TFLA, probiotics or the synbiotic, the log-rank test revealed significant variations between the transgenic lines treated with either Lf5221 or probiotic (*p*<0.05), but not with TFLA or synbiotic (*p*<0.05) (S2 Fig), which is consistent with the median survivability analysis observed in Fig 1.

Motility was assessed using the negative geotaxis test as an indicator of metabolic health in aging flies. In untreated APP-BACE1 controls, there was a time-dependent decrease in motility demonstrated by an 87% decrease in climbing ability by day 30 (Fig 1b; p < 0.01). At day 30, the probiotic group showed a significant improvement in motility compared to the Lf5221 group, while the synbiotic group showed significant improvement compared to all of the treatment groups. As a marker of AD, the accumulation of Aβ was assessed from *Drosophila* head extracts. In controls, a 30% increase in Aβ levels was observed at day 30 compared to young untreated flies, an elevation that was significantly decreased by the TFLA, probiotic and synbiotic groups at day 30 (Fig 1c; p < 0.01). Notably, the probiotic and synbiotic formulations at day 30 rescued Aβ to the level of day 0 controls although demonstrated an intriguing pattern of elevated Aβ expression at days 10 and 20, which would require further investigation. Acetylcholinesterase (ACh) activity, as expected, was likewise decreased in aging AD controls by almost 50% (Fig 1d). By day 30, TFLA, the probiotic and synbiotic groups rescued ACh activity with the synbiotic formulation having a higher impact on ACh activity than Lf5221 alone.

**Fig 2:**
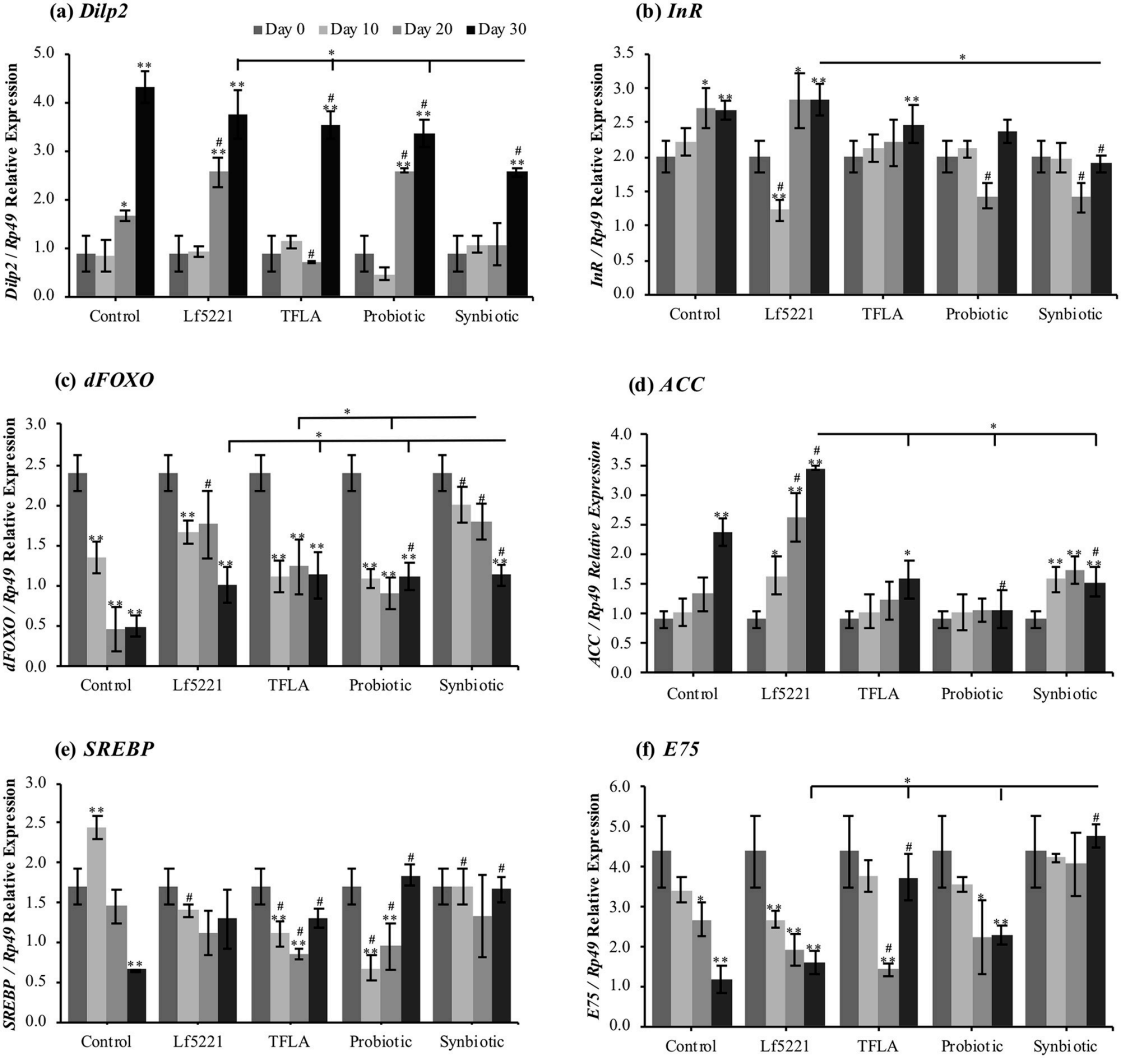
Genetic markers of insulin resistance and fatty acid metabolism are differentially affected by probiotic and/or prebiotic treatment in AD *Drosophila*. Transgenic AD (APP-BACE1) *Drosophila melanogaster* raised on untreated (control) media, or media supplemented with Lf5221, TFLA, the probiotic or synbiotic formulation were sampled at days 0, 10, 20 and 30 and prepared for RNA extraction and gene expression analysis. Genetic markers of insulin resistance included (a) *Drosophila insulin-like peptide (Dilp)*2, (b) Insulin receptor (InR) and (c) *Drosophila Forkhead Box O* (*dFOXO*) while genetic markers of fatty acid metabolism included (d) acetyl co-A carboxylase (ACC), (e) sterol regulatory element binding protein (SREBP) and (f) the PPARγ downstream target E75. Each group is the average of n = 5 independent groups +/- SEM. Significance is indicated as black stars (*) relative to the day 0 control * p < 0.05 and ** p < 0.01 or hash (#) relative to the non-supplemented control at the same time point where # indicates p < 0.05.

Metabolic stress is a major risk factor to the onset and aggravation of AD so the impact of the probiotic, prebiotic and combinatorial therapy on a variety of metabolic markers was assessed in aging AD *Drosophila*. Total glucose levels were elevated by almost 60% at day 30 in untreated APP-BACE1 controls and remained elevated in all groups except those receiving the synbiotic treatment, which had no change in glucose levels at any age (S3 Fig). Variations in total glucose were reflected by the expression of the canonical insulin-signaling factors. Gene expression of *Drosophila insulin-like peptide* (*dilp*)2 was elevated in all groups at day 30, though, compared to controls, reduced by the TFLA, probiotic and synbiotic formulations with the synbiotic having a greater impact than the other treatment groups (Fig 2a). *Dilp*3 had a similar effect, though was only reduced by the synbiotic formulation at day 30 (S3 Fig). Expression of InR was likewise elevated in controls at days 20 and 30, with a complete rescue by the probiotic and synbiotic formulations (Fig 2b). There were similar changes in the downstream insulin signaling cascades measured with dAkt (S3 Fig), dTor (S3 Fig) and dFOXO (Fig 2c). In particular, dFOXO expression was significantly reduced over time in the control group reflecting functional insulin resistance with only the synbiotic formulation eliciting beneficial effects on dFOXO expression over all time points and the probiotic formulation eliciting a beneficial effect at day 30.

The level of total triglycerides was similarly elevated in aging control *Drosophila* yet was only reduced at day 30 by the probiotic and synbiotic formulations (S4 Fig). This increase in total triglycerides was reflected by increased gene expression of *acetyl CoA carboxylase* (ACC) at day 30 in untreated APP-BACE1 controls, which was subsequently reduced by treatment with both the probiotic and synbiotic formulations at day 30 (Fig 2d). *Fatty acid synthase* (FAS) gene expression was also elevated in controls at day 30 and rescued by TFLA, the probiotic and synbiotic treatments (S4 Fig). Sterol regulatory element binding protein (SREBP) is the transcriptional regulator of ACC and FAS, however in contrast, its gene expression was reduced in a time-dependent manner in controls and rescued by all treatment regimens (Fig 2e). Nevertheless, a decrease in the gene expression of the gluconeogenesis factor phosphoenol pyruvate carboxykinase (PEPCK) was observed over time in controls, elevated only by the synbiotic formulation (S4 Fig) whereas the reduced gene expression of lipid storage droplet (LSD)2 in controls was elevated by both TFLA and synbiotic treatment (S4 Fig). Finally, peroxisome proliferator activated receptor (PPAR)γ is a broad transcriptional regulator controlling lipogenesis and adipogenesis. In *Drosophila*, E75 is the direct downstream target of PPARγ and as expected, E75 expression was reduced over time in control AD flies (Fig 2f). Interestingly, TFLA treatment increased E75 expression at day 30 while the synbiotic formulation completely rescued E75 expression at all time points attributing PPARγ regulation to the positive beneficial effects of the synbiotic formulation on metabolic stress in the context of AD.

**Fig 3:**
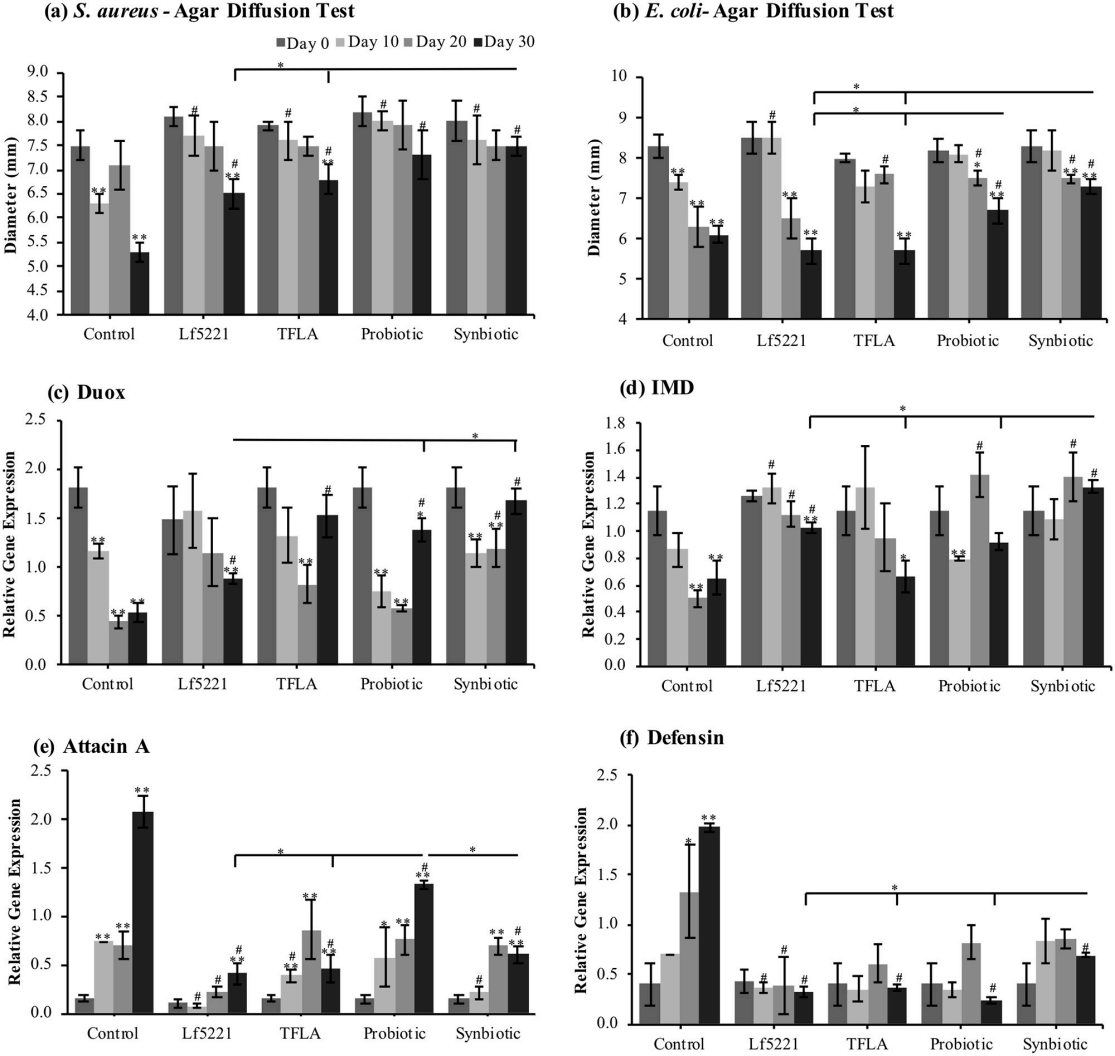
Inflammatory markers are reduced by the synbiotic formulation in aging AD *Drosophila melanogaster*. The level of circulating antimicrobial agents produced by AD *Drosophila* reared on untreated media (controls) or supplemented with either Lf5221, TFLA, the probiotic or synbiotic formulation was tested using the agar diffusion test on either media supplemented with (a) *S*. *aureus* or (b) *E*. *coli* species. The level of antimicrobial production is measured as the diameter of the zone of inhibition. The inherent level of the innate immune response in *Drosophila* was assessed by examining the gene expression of key immune mediators over time including (c) *Dual oxidase* (*Duox*), (d) *Immune deficiency* (*IMD*) and the antimicrobial peptides (AMPs) (e) *Attacin A* and (f) *Defensin*. Each group is the average of n = 5 independent groups +/- SEM. Significance is indicated as black stars (*) relative to the day 0 control * p < 0.05 and ** p < 0.01 or hash (#) relative to the non-supplemented control at the same time point where # p < 0.05.

Inflammaging, or the gradual increase in inflammation due to age without specific immune aggregates, is a major risk factor in AD. To test the efficiency of the AD *Drosophila* immune system following supplementation with synbiotics, hemolymph was extracted and placed on agar plates coated with either the gram-positive pathogen *S*. *aureus* or the gram-negative pathogen *E*. *coli* where through the agar diffusion test, the zone of inhibition directly represents the quantity of circulating antimicrobial factors. The zone of inhibition on plates coated with *S*. *aureus* treated with control hemolymph decreased by almost 30% by day 30 (Fig 3a). This decrease was significantly elevated by all of the treatment regimens with the synbiotic group having a significantly greater effect than either Lf5221 or TFLA alone. Considering *E*. *coli*, the zone of inhibition was similarly decreased by 27% at day 30 in controls (Fig 3b) though was only ameliorated by treatment with either the probiotic or synbiotic formulations.

Investigating the impact of the probiotic and synbiotic treatments on the specific innate immune factors in *Drosophila*, gene expression of a variety of immunological factors was determined in aging AD *Drosophila*. Gene expression of the indiscriminate innate immune factor *dual oxidase* (*Duox*) which normally responds to elevated uracil levels from pathogenic bacteria, decreased over time in aging controls with a maximal decrease by 70% at day 30 (Fig 3c). At day 30, treatment with Lf5221, the probiotic or synbiotic formulations increased *Duox* expression, with the level achieved by the probiotic or synbiotic formulation being significantly higher than Lf5221. Immune deficiency (*IMD*) expression, induced in response to gram-negative and some gram-positive bacteria, was similarly reduced by almost 60% at day 30 in controls (Fig 3d). By day 20, all treatment groups had a positive effect on *IMD* gene expression while at day 30, only Lf5221 and to a greater extent the synbiotic formulation significantly elevated *IMD* expression. *Relish*, a cytokine-like immune mediator activated downstream of IMD, was not varied in the control group although there were significant reductions by TFLA and the probiotic formulation at days 10, 20 and 30 and the synbiotic treatment at day 10 (S5 Fig).

The antimicrobial peptides (AMPs) are a set of humoral immune mediators in *Drosophila* targeting different types of immunological insults. *Attacin A* targets both gram-positive and gram-negative bacteria and in controls upregulated gene expression at days 10 through 30 to a maximum amount of almost 14-fold. By day 30, *Attacin A* gene expression was reduced by all treatment groups to similar extents (Fig 3e). *Defensin*, acting downstream of gram-positive immune insults via the Toll pathway, was similarly upregulated in controls by day 30 and this increase was rescued by all treatment groups at all time-points (Fig 3f). Finally, *Diptercin*, responsive to gram-negative bacterial insults downstream of IMD signaling, was significantly upregulated in control AD *Drosophila* at days 10 through 30, which was reduced to similar extents by each of the treatment groups (S5 Fig).

**Fig 4:**
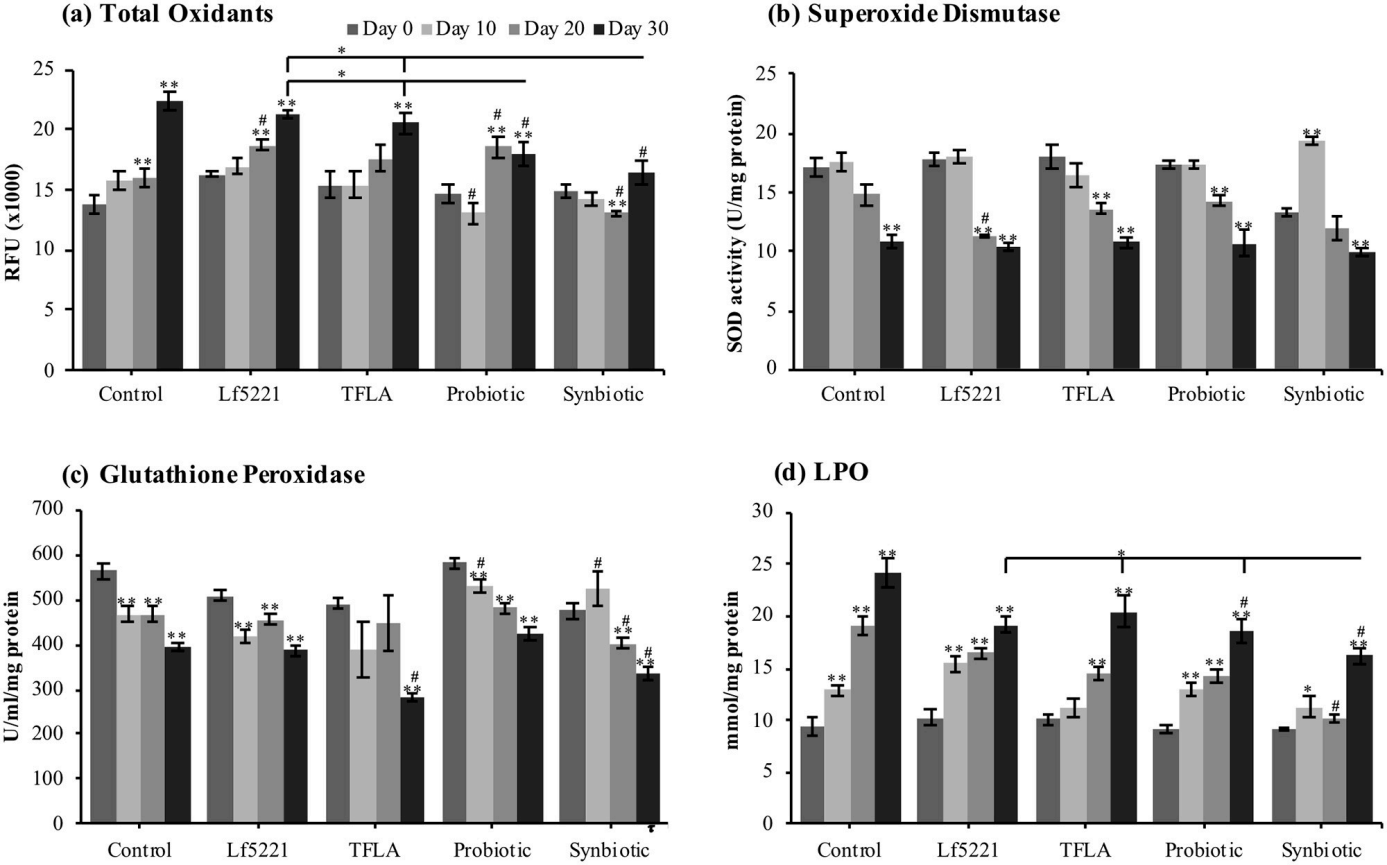
Variations in oxidative stress markers in AD *Drosophila melanogaster* supplemented with a synbiotic formulation. Oxidative stress was measured in *Drosophila melanogaster* raised on untreated media (controls) or media supplemented with Lf5221, Triphala (TFLA), the probiotic or synbiotic formulation at days 0, 10, 20 and 30. Oxidative stress was assessed by quantifying (a) total oxidants, (b) superoxide dismutase (SOD) activity, (c) glutathione peroxidase (GPx) activity and (d) lipid peroxidation (LPO) prevalence. Each group is the average of n = 5 independent groups +/- SEM. Significance is indicated as black stars (*) relative to the day 0 control * p < 0.05 and ** p < 0.01 or hash (#) relative to the non-supplemented control at the same time point where # p < 0.05.

According to the most popular theory of aging, oxidative stress accumulates with age and like inflammation, aggravates symptoms of AD. In aging AD *Drosophila*, the level of total oxidants increased at days 20 and 30, with the latter having a 63% increase compared to day 0 (Fig 4a). Only the probiotic and synbiotic treatments at day 30 showed significant reductions in total oxidants compared to controls, which were significantly lower than the Lf5221 and TFLA groups. Superoxide dismutase (SOD) activity (Fig 4b) and glutathione peroxidase (Fig 4c) were both reduced over time in the control groups; however there were few variations in their activity following probiotic and/or prebiotic treatment. Finally, lipid peroxidation (LPO) expression was highly upregulated in controls over time, peaking at day 30 with a 2.6-fold increase (Fig 4d). The probiotic and synbiotic formulations significantly reduced LPO levels at day 30, with the synbiotic formulation having a greater impact than the probiotic formulation, TFLA or Lf5221.

**Fig 5:**
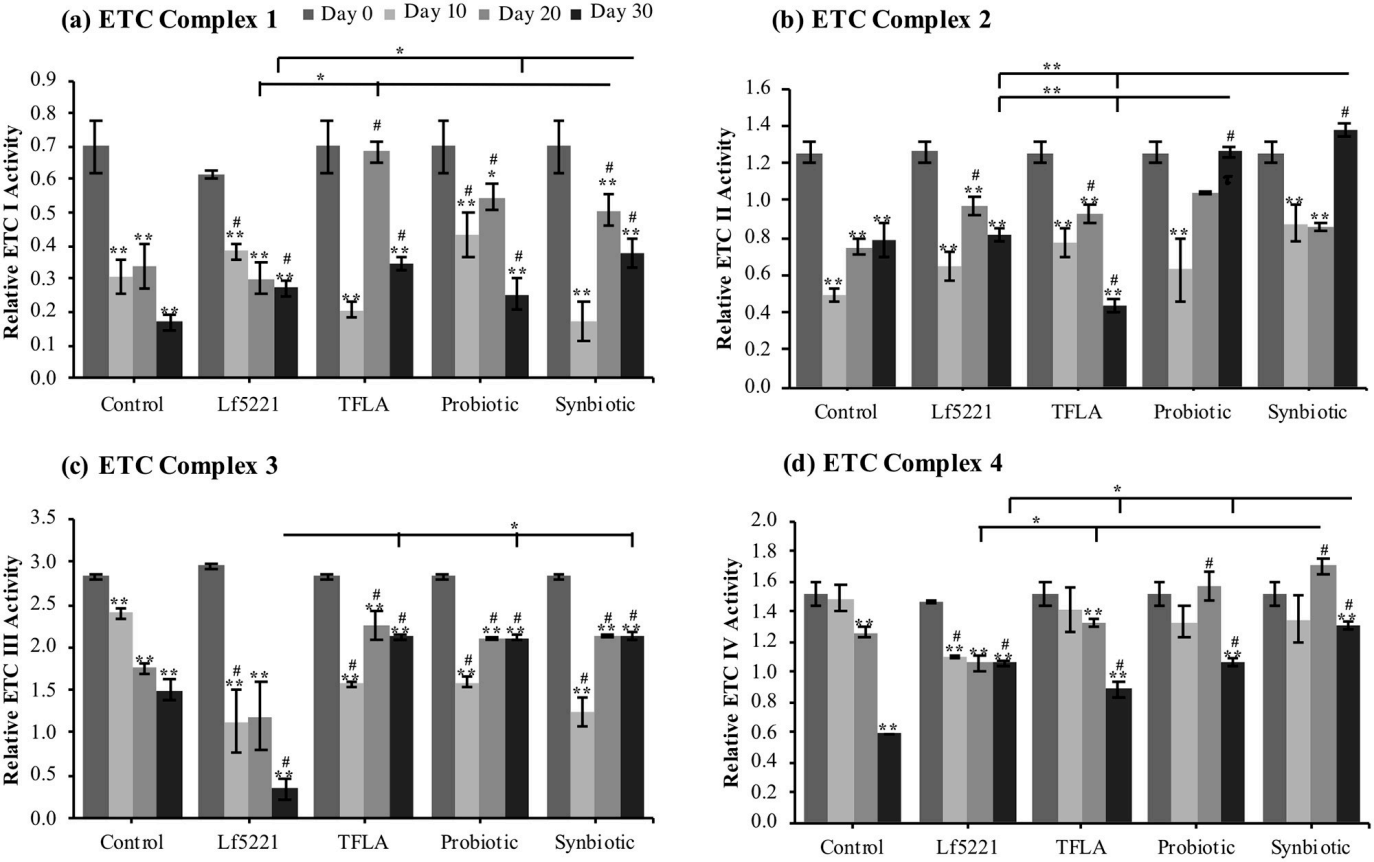
Electron transport chain activities are beneficially affected by synbiotic treatment in AD *Drosophila melanogaster*. Activity of each of the individual electron transport chain (ETC) complexes was assessed in AD *Drosophila melanogaster* supplemented with either Lf5221, TFLA, the probiotic or synbiotic formulations. Activity levels of (a) complex 1 (NADH coenzyme Q reductase), (b) complex 2 (succinate dehydrogenase), (c) complex 3 (cytochrome bc1 complex) and (d) complex 4 (cytochrome c oxidase) were assessed with various colourmetric assays. Each group is the average of n = 5 independent groups +/- SEM. Significance is indicated as black stars (*) relative to the day 0 control * p < 0.05 and ** p < 0.01 or hash (#) relative to the non-supplemented control at the same time point where # p < 0.05.

Since there were reductions in the accumulation of oxidants, but not in the level of activity of the antioxidant enzymes SOD and GPx, the activity of the ETC complexes was assessed to determine how the prebiotic and probiotics are impacting the production of oxidants through mitochondrial metabolism. NADH coenzyme Q reductase (ETC complex I) activity was decreased by 78% in controls by day 30 (Fig 5a). Treatment with TFLA, the probiotic and synbiotic formulations elevated ETC complex I activity by day 20, with the probiotic and synbiotic formulations having the greatest impact at day 30. Succinate dehydrogenase (ETC complex II) activity was reduced over time in controls peaking at a 38% loss of activity by day 30, which was only improved following probiotic or synbiotic treatment (Fig 5b). Cytochrome bc_1_ complex (ETC complex III) activity showed time-dependent reductions in controls peaking at almost 50% loss of activity by day 30 (Fig 5c). TFLA, the probiotic and synbiotic formulations all improved ETC complex III activity at days 20 and 30. Finally, cytochrome c oxidase (ETC complex IV) activity was reduced in controls by 62% at day 30, an effect improved by all treatment groups but most significantly by the synbiotic formulation at both days 20 and 30 (Fig 5d).

**Fig 6:**
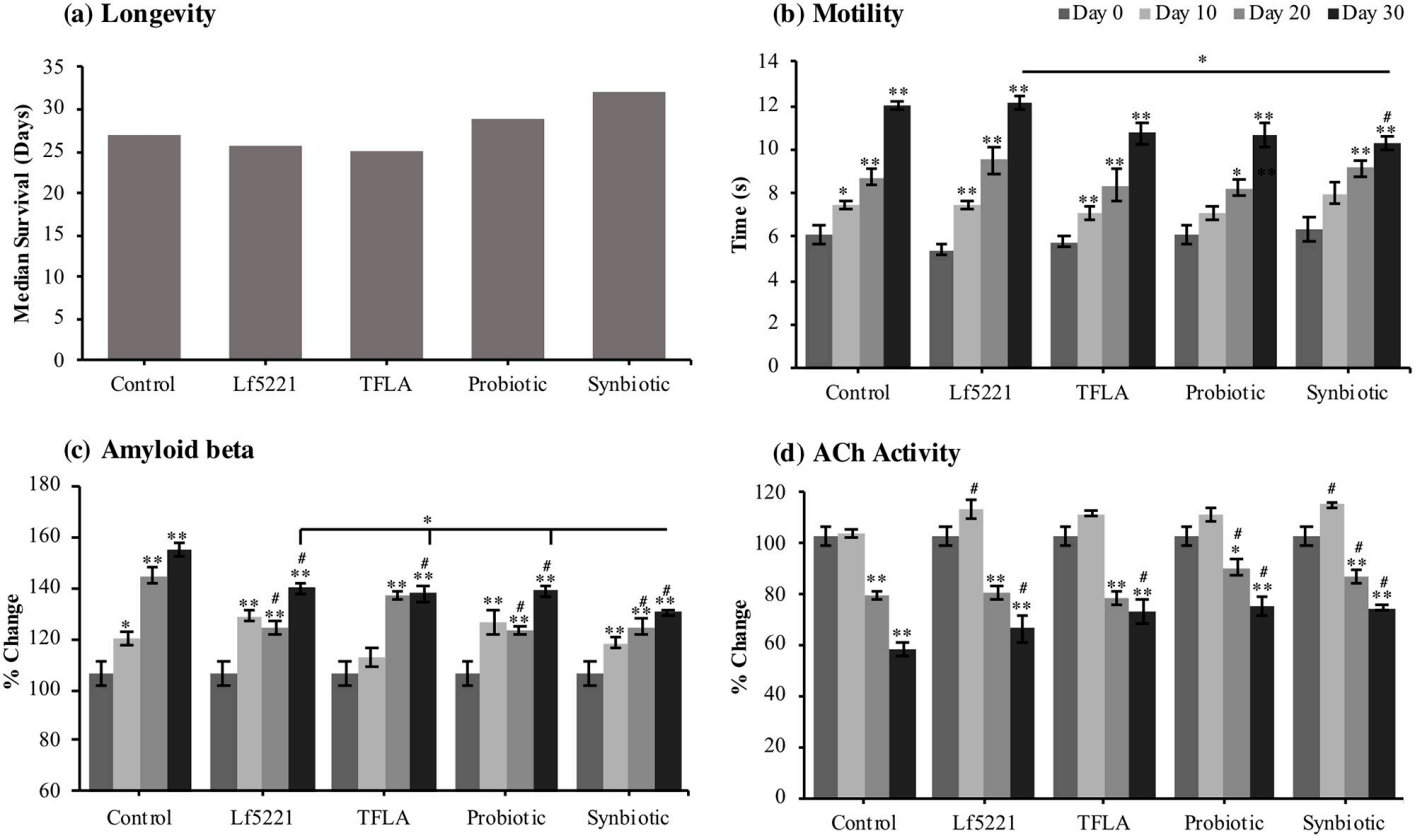
BADGE treatment in *Drosophila melanogaster* attenuates markers of AD despite synbiotic supplementation. To test the contribution of PPARγ activity in the synbiotic formulation’s action in pathological markers of AD, *Drosophila melanogaster* supplemented with the Lf5221, TFLA, the probiotic or synbiotic formulation were co-treated with bisphenol A diglycidyl ether (BADGE), a PPARγ antagonist. After treatment, (a) longevity, (b) motility, (c) amyloid beta (Aβ) accumulation and (d) acetylcholinesterase (ACh) activity was assessed. Each group in b, c and d is the average of n = 5 independent groups +/- SEM where the lifespan analysis is the result of a single lifespan analysis. Significance is indicated as black stars (*) relative to the day 0 control * p < 0.05 and ** p < 0.01 or hash (#) relative to the non-supplemented control at the same time point where # p < 0.05.

It was demonstrated in the assessment of metabolic marker expression that of the *Drosophila* PPARγ target E75 was significantly reduced over time in control AD *Drosophila*, which was rescued by treatment with the synbiotic formulation. PPARγ has broad implications in the regulation of metabolism and energy homeostasis and has been identified as a player in the pathogenesis of AD mostly through the regulation of metabolic stress in neurons. However, PPARγ also plays significant roles in inflammatory mechanisms and consequently, oxidative stress. To test the hypothesis that the action of the synbiotic formulation is working through modulation of PPARγ activity, *Drosophila* were treated with a specific antagonist of PPARγ: bisphenol A diglicydyl ether (BADGE). Longevity and motility of BADGE-treated AD *Drosophila* supplemented with the prebiotics and/or probiotics were dramatically altered compared to non-BADGE treated flies. Using the log-rank test, no significant variations in longevity between the control and prebiotic and/or probiotic treated *Drosophila* were observed (S6 Fig) as evident with the median survival assessment (Fig 6a). In addition, BADGE treatment eliminated any beneficial effect of treatment on motility in the aging AD *Drosophila* (Fig 6b). The synbiotic formulation’s impact on Aβ expression was also negatively affected by BADGE treatment. Where the synbiotic formulation rescued Aβ levels at days 20 and 30 in untreated *Drosophila*, BADGE treatment significantly attenuated the beneficial effect of the synbiotic treatment on Aβ expression in AD *Drosophila* (Fig 6c). Finally, the reduced ACh activity variations at days 20 and 30 in controls which were ameliorated by the prebiotic and probiotic treatments in untreated AD flies, were similarly blunted by the BADGE treatment (Fig 6d). This indicates that transcriptional regulation of PPARγ is implicated by the prebiotic and probiotic formulations which has therapeutic for the management of AD.

Metabolic markers were also assessed in BADGE-treated *Drosophila*. The 2.19-fold increase in total glucose from days 0 to 30 in BADGE-supplemented controls was not reduced by any of the treatment groups (S3 Table). Similarly, variations between time and group in *dilp*2, *dilp*3 and *InR* mRNA expression were abolished by BADGE treatment. Distinct changes in the dAkt-dTOR-dFOXO signaling cascade were also observed following BADGE-supplementation. *dAkt* expression in control BADGE-supplemented AD *Drosophila* was reduced by 0.18-fold at day 30, which was slightly upregulated by TFLA and the probiotic formulation. Conversely, *dTOR* was strongly upregulated by 2.45-fold in controls and reduced by all treatment groups except the synbiotic formulation. Finally, there was no change in *dFOXO* expression over time except in the synbiotic group which demonstrated a slight elevation of *dFOXO* at day 30 (S3 Table).

The 1.82-fold elevation in total triglycerides in BADGE-treated controls over time was reduced by Lf5221, TFLA and the probiotic formulation with no effect by the synbiotic treatment. This was accompanied by a 1.58-fold elevation of *ACC* and a 1.52-fold elevation in *FAS* mRNA expression in control groups. *ACC* was also downregulated by all treatment groups while *FAS* was only slightly elevated by the probiotic formulation. *PEPCK* mRNA expression was unaffected in controls and no change was observed in any of the treatment groups. *Lsd2* and *SREBP* mRNA expression were significantly reduced in BADGE-supplemented controls at day 30, with no change elicited by prebiotic and/or probiotic treatments. Finally, as expected, there was a significant downregulation of E75 at day 30 in controls and this expression remained very low in all treatment groups, except the synbiotic group which demonstrated a slight increase in E75 expression at day 30 (S4 Table).

Finally, BADGE supplementation abolished any effect of the prebiotic and/or probiotic treatment on the agar diffusion test for both *S*. *aureus* and *E*. *coli* indicating that PPARγ-mediated transcription modulated by the treatment groups has an important impact on immune regulation in aging AD *Drosophila* (S5 Table). The reduction of *Duox* and *IMD* expression was also generally unaffected by any prebiotic or probiotic treatment in BADGE-treated AD *Drosophila*. *Relish* mRNA expression remained unchanged in control *Drosophila* though was slightly elevated by Lf5221, the probiotic and synbiotic formulations. Each of the tested AMPs showed a slight elevation over time in the control BADGE-supplemented AD *Drosophila*; however, there was no consistent change to their expression by any of the treatments.

## Discussion

AD is an intricate age-related chronic disease whose pathogenesis cannot be attributed to any single cause. Neuroinflammation, metabolic instability, elevated oxidative stress and imbalanced neurochemical signaling all contribute to the development of AD and due to the heterogenous nature of these risk factors, no isolated pharmaceutical agent is capable of simultaneously targeting all risk factors to effectively halt disease progression. Likewise, current therapies for AD only treat isolated disease symptoms including Aβ and tangle accumulation, memory loss, imbalances in ACh levels, behavioural changes or sleep changes [24]. Some of the major breakthroughs for understanding AD pathology comes from the repositioning of existing therapeutic compounds towards their therapeutic potential in AD. These include therapies used for T2D in addition to angiotensin receptor blockers, retinoid therapy and minocycline [24] however, these and other AD therapies have shown only minor and inconsistent successes.

**Fig 7:**
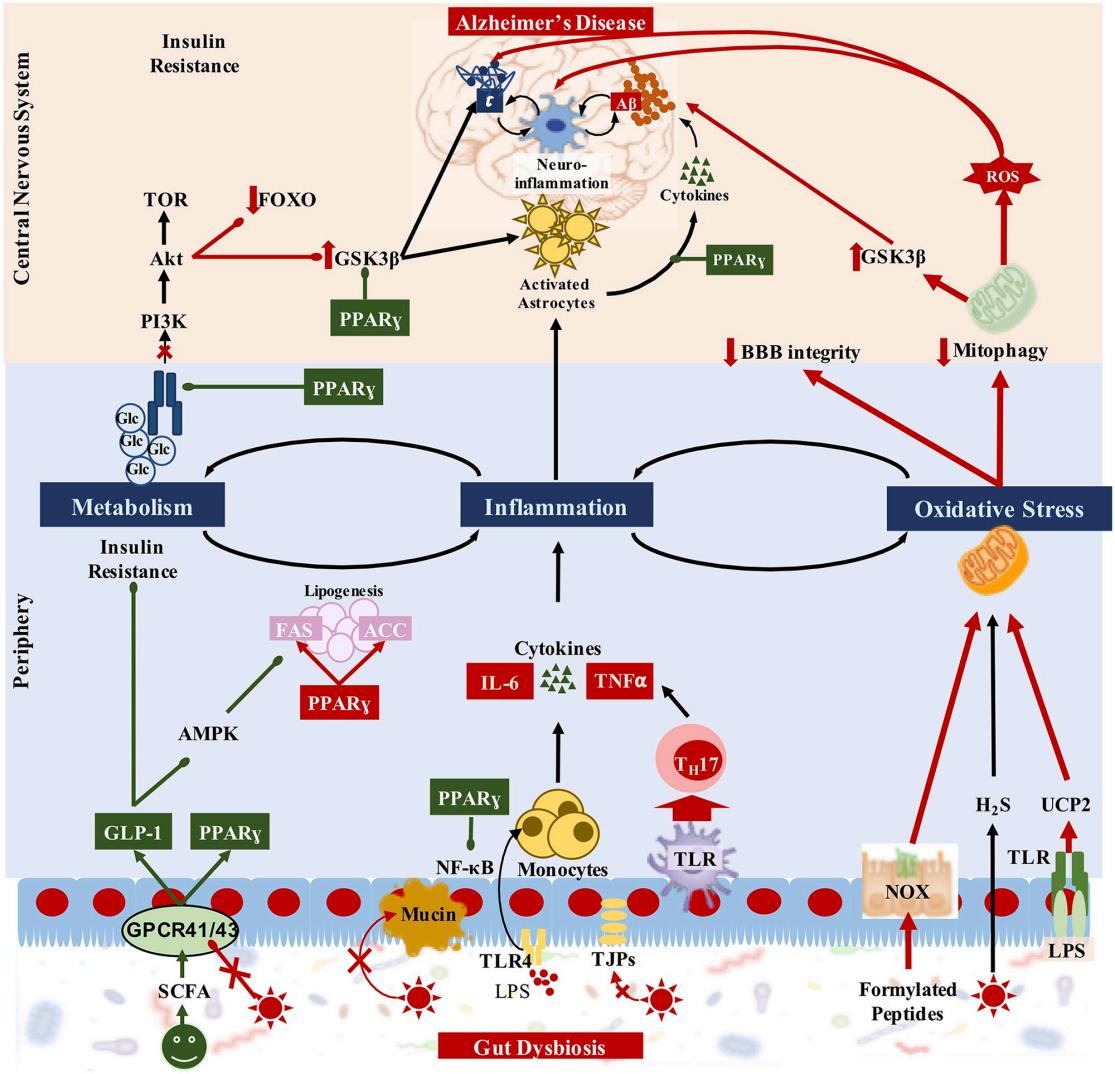
Proposed mechanisms of PPARγ interference with gut-brain-axis communication in the context of Alzheimer’s disease. Metabolism, inflammation and oxidative stress are three physiological states whose integrated pathology forms the main stochastic risk factors of Alzheimer’s disease. The gut microbiota and its associated metabolites influence each of these axes through several mechanisms as depicted here. Abbreviations: GPR—G-protein receptor; SCFA—short chain fatty acids; GLP-1—glucagon like protein; PPAR—peroxisome proliferator activated receptor; TLR—toll like receptor; TJP—tight junction protein; IL-6; interleukin-6; TNF—tumor necrosis factor; NOX—NADPH oxidase; H_2_S—hydrogen sulfite; UCP2—uncoupled protein 2; LPS—lipopolysaccharide; GSK3β—glycogen synthase kinase 3 beta; PI3K—phosphoinositide-3 kinase; FOXO—Forkhead box protein O; Akt—protein kinase B; ROS—reactive oxygen species; BBB—blood brain barrier.

The current study describes a novel next-generation AD therapy that effectively targets multiple aspects of AD’s stochastic pathology by utilizing mechanisms of the GBA potentially through the broad-acting transcriptional regulatory PPARγ (Fig 7). The synbiotic formulation utilized in this study is a combination of three probiotic bacteria together with a novel plant-derived polyphenol-rich prebiotic TFLA [15]. This formulation was previously shown to activate the metabolic, anti-inflammatory, anti-oxidative and mitochondrial regulatory axes [25] through mechanisms of GBA communication and in the present study, these effects were extended for its potential therapeutic use in AD. Notably, the synbiotic formulation, if considered in each individual risk factor of AD, does not necessarily have a greater impact than its individual components; however, when multiple risk factors of AD are assessed, the synbiotic is the only formulation which has consistent beneficial effect across these multiple risk factors making the synbiotic a better choice at managing the heterogeneity of AD’s etiology. Considering that AD is inherently a multifaceted disease, the synbiotic formulation provides a more comprehensive treatment paradigm compared to isolated pharmacological strategies.

Survivability in control AD *Drosophila* was significantly elevated by the TFLA, probiotic formulation and most significantly, the synbiotic formulation. The increase in survival was accompanied by an equal improvement in motility and similar improvements in Aβ accumulation and ACh activity. Interestingly, the probiotic and synbiotic formulations, despite having significant variations in survivability, had similar effects on Aβ accumulation and ACh activity indicating that these canonical AD pathological markers are not the only measure of mortality in AD but the stochastic environmental contributors also have a significant impact.

A few recent studies have indicated that probiotic treatment may reduce the pathological markers of AD, but none have been as successful as the synbiotic formulation presented here. In one study, *L*. *plantarum* MTCC 1325 in AD albino rats reduced memory impairment, rescued ACh levels and reduced Aβ plaques [26]. The *B*. *breve* strain A1 prevented cognitive dysfunction in AD mice while suppressing hippocampal expression of inflammatory and immune-reactive genes induced by Aβ [27]. Another study in 3xTg-AD mice showed that the probiotic formulation SLAB51 containing 9 bacterial strains influenced plasma cytokine levels, metabolic hormones, autophagy, cognitive decline and amyloid aggregates [28]. However, a clinical study revealed that a probiotic cocktail had no effect on oxidative stress or inflammatory biomarkers, though did positively affect cognitive function and metabolic status of AD patients [27]. Clearly, the composition of the probiotic formulation is critical for determining its effectiveness against AD.

Polyphenols have been shown to have potent benefits against AD by targeting Aβ neuropathology, neuroplasticity, neuroinflammation, tau fibrillation and providing anti-oxidant protection [29]. One example is grape seed polyphenol extracts which, through modifications of the gut microbiota, inhibit Aβ oligomerization and reduce cognitive impairment in Tg2576 mice [30, 31]. Dietary polyphenols are consumed in their most active form nor are they able to cross the BBB, so their biotransformation by the gut microbiota is essential to create bioactive metabolites which are responsible for their disease-altering activity. For example, the red wine polyphenol biotransformation product quercetin-3-O-glucoside is capable of modulating Aβ neuropathogenic mechanisms while 3’-O-methyl-epicatchin-5-O-β-glucuronide was shown to modulate synaptic plasticity and the bioavailability of both of these transformations are dependent on the gut microbiota [32].

The present formulation has several benefits that contribute to its success as an AD therapy. TFLA contains a high concentration of gallic acid, chebulinic acid and epicatechin [33]. Gallic acid inhibits Aβ aggregation and amyloid fibrils in vitro [34] and in a rat model of AD, gallic acid was shown to reduce neural damage and amyloid neuropathology by increasing free radical scavenging and inhibiting Aβ oligomerization [35]. Lf5221 is a potent producer of ferulic acid (FA), a hydroxycinnamic acid with anti-oxidant, anti-inflammatory and anti-AD activity [36]. The activity of FA is dependent on the esterase activity of Lactobacillus bacteria, including Lp8826, which converts FA into 4-vinylguaiacol and hydroferulic acid [37] and eventually into its final bioactive metabolic products caffeic and vanillic acid. FA and its metabolites have several benefits as an anti-AD therapeutic including anti-inflammatory, anti-oxidant, anti-apoptotic effects and inhibition of Aβ fibril formation [38]. As insinuated, vanillic acid also has neuroprotective abilities by reducing oxidative damage and neuroinflammation in a Aβ1–42 induced AD model [39].

Metabolic dysfunction, especially insulin resistance, is a key risk factor in the early development of AD [40] and several attempts have been made to develop pharmaceutical treatments for AD by managing insulin signaling such as an intranasal insulin spray and GLP-1 agonists. In the present model, the synbiotic formulation significantly reduced the accumulation of glucose and insulin resistance while ameliorating variations in the downstream signaling cascades indicating a multi-level action on metabolic signaling, along with several other biological effects.

Chronic neuroinflammation without leukocyte infiltration is one of the key risk factors of AD progression [41]. Activated astrocytes are recruited to the sites of Aβ plaques where they secrete inflammatory cytokines and inflammatory enzymes exasperating the AD phenotype. Long-term use of NSAIDs decreases AD development, slows disease progression and improves cognitive functioning [42]; however, in more involved clinical trials, the therapeutic efficacy of NSAIDs was limited [43]. Inflammation was present in the current model reflected by the reduction of secretable antimicrobial factors as demonstrated in the agar diffusion assays and expression of the innate immune system genes. In both cases, the probiotic and synbiotic formulations had combinatorial effects on immune efficacy. While the probiotic formulation demonstrated beneficial effects, the synbiotic formulation rescued the antimicrobial production and the expression of *Duox* and *IMD*, indicating that the addition of TFLA is essential to maximize the anti-inflammatory impact of the probiotic formulation. The gut microbiota is absolutely critical for mounting an immune response as 80% of the genes induced following septic injury in *Drosophila* are downstream of the IMD and Toll pathways and activated by microbial ligands [44]. In addition, the gut microbiota is critical for management of gut epithelial integrity through maintenance of tight junction proteins and mucin production therefore inhibiting the infiltration of bacterial cells and products that would induce an immune response.

Oxidative stress, like inflammation, is a fundamental risk factor for the development and progression of AD. LPO is greatly enhanced in AD and is formed when ROS particles attack lipids in the cell membrane to create reactive lipid species that are crosslinked and difficult to eliminate [45]. Several antioxidant therapies have been tested in AD patients with little success due to their inability to cross the blood-brain-barrier [46, 47]. In the current study, there was a clear increase in total oxidants and lipid peroxidation in the aging AD *Drosophila* and decreased activity of the antioxidant enzymes SOD and GPx. The probiotic and to a greater extent the synbiotic formulation effectively lowered total oxidant and LPO levels; however the synbiotic formulation did not improve the activity of either SOD or GPx indicating that its anti-oxidant activity is derived from its ability to reduce ROS production instead of increasing the capacity to recycle ROS.

Mitochondria is the major source of oxidative stress in the body due to the leakage of electrons during electron transfer which increases with age. Mitophagy and the fission and fusion cycles of mitochondria in AD are dysregulated leading to structurally damaged swollen mitochondria contributing to oxidative stress [48]. In the present study, the activity of all the key ETC complexes were significantly reduced over time in untreated AD *Drosophila*, a phenomenon that has been reported in other studies and correlated with clinical state and plaque counts [49]. Importantly, both the probiotic and synbiotic formulations increased the activity of all the ETC complexes while Lf5221 and TFLA alone only positively affected complexes 1 and 4. This supports the previous observation that the probiotic and synbiotic formulations reduce oxidative stress by preventing ROS production rather than ameliorating their accumulation.

It is clear that the gut microbiota has a significant influence on several pathological states that influence the development and progression of AD; however, it is less obvious how the gut microbiota is mediating this communication. The gut microbiota produces many metabolic byproducts, bioactive molecules and endocrine mediators that can communicate with the host [9]. In addition, through the interaction of their surface moieties with the host cells, various intracellular cascades are initiated. The molecular communication from the current formulation is likely multi-faceted due to the formulas’ complexity; however, one intermediate transcription factor, PPARγ, has been shown in preceding studies to implicate GBA communication in management of metabolism [25].

PPARγ is a lipid sensor, binding to fatty acids, eicosanoids and other natural lipids to regulate the deposition of fatty acids in adipose tissue. The role of PPARγ in the regulation of metabolism and glucose homeostasis has been widely studied as PPARγ indirectly improves insulin sensitivity and enhances glucose disposal through the regulation of free fatty acids [50]. PPARγ also has a heavy presence in astrocytes and microglia where it induces production of anti-inflammatory gene-related expression and downregulates proinflammatory mediators by suppressing NK-kB, AP-1 and STAT-1 in activated microglia and macrophages [51]. In the brain, PPARγ activation may directly influence neuron cell viability and differentiation [52]. Presence of a particular allele of PPARγ, Ala12, is correlated with octogenarian AD patients [53] instigating an almost 2-fold increased risk of developing AD. PPARγ expression also directly impacts Aβ homeostasis and immunostimulated BACE1 expression is silenced by PPARγ-transcriptional inhibition [54]. Oral pioglitazone (a PPARγ agonist) reduced BACE1 transcription in APP transgenic mice while increasing Aβ clearance in both glia and neurons [55]. Oral rosiglitazone treatment reduced Aβ_1–42_ by 25% without affecting Aβ_1–40_ levels [56] while in a Tg2576 AD mouse model, reduced cognitive decline and aberrant neuronal activity in the dentate gyrus [57]. The problem with current PPARγ therapies is they have potent side effects in cardiovascular health and weight gain due to the significant involvement of PPARγ in metabolic regulation.

In the present study, inhibition of PPARγ activity with the antagonist BADGE almost eradicated the beneficial effects on AD pathology elicited by all the treatment groups. This universal effect indicates that PPARγ is a significant player in the GBA communication, either directly or indirectly, and can be among the clinical targets of future probiotic or synbiotic therapies in the management of chronic multifactorial diseases such as AD. Interestingly, it has been observed that the protective effect of SCFAs on the high-fat diet-induced metabolic alterations seems to be dependent on down-regulation PPARγ indicating that the production of SCFAs from the gut microbiota may be a key mechanism of communication with PPARγ activity. In particular, butyrate causes the expansion of the PPARγ coactivator PGC-1α in several tissues [58] and was also shown to upregulate PPARγ in Caco-2 cells [59]. Interestingly, PPARγ has been shown to modulate BACE transcriptional regulation, providing a possible direct link between PPARγ regulation and AD [54]. The implications of this study demonstrating the role of PPARγ in the probiotic- and synbiotic-mediated benefits in AD is that it expands the potential mechanisms of GBA communication and provide a potential clinical targets that could be relevant in chronic diseases that can be modulated by the gut microbiota. Importantly, despite BADGE treatment, the synbiotic formulation was able to stimulate some E75 transcription indicating that the bioactive metabolites produced by the synbiotic formulation may have a greater ability to stimulate PPARγ activation than the probiotic or TFLA alone. In addition, the synbiotic formulation also had a positive impact on the downstream *dTOR* and *dFOXO* mRNA expression despite BADGE treatment again indicating that there are some additional activities provided by the synbiotic treatment, possibly due to its putative increase in bioactive metabolites from the addition of TFLA, with biological activity against independent AD risk factors. This could explain, in part, why the synbiotic formulation has greater therapeutic efficacy than the probiotic or TFLA supplementation alone.

AD is a multi-faceted disease that is largely caused by the accumulation of environmental stresses to which the body loses its ability to fight with age. The synbiotic formulation presented in the current study represents a novel next-generation therapeutic option for AD that not only reduces the canonical markers of AD including premature mortality, Aβ accumulation and loss of ACh activity, but also ameliorates irregularities in metabolism, immune and oxidative stress signaling. For the first time, a probiotic formulation has shown potential as an inclusive AD therapy that simultaneously and combinatorially targets multiple aspects of AD pathogenesis. In addition, the polyphenol-rich supplement TFLA adds a boost of polyphenolic bioactive products that promotes all the beneficial effects of the probiotic formulation while balancing the overall population of the gut microbiota. Overall, the use of optimized next-generation synbiotic formulations provides a paradigm shift for AD treatment by providing a more effective, sustainable and affordable preventative therapy without harmful side effects.

## Supporting information

S1 Table Primer sequences to identify various metabolic markers in *Drosophila*. (DOCX)

S2 Table Primer sequences of *Drosophila melanogaster* inflammatory markers. (DOCX)

S3 Table Glucose metabolic markers in AD *Drosophila melanogaster* co-treated with probiotics and/or prebiotics with BADGE. (DOCX)

S4 Table Fatty acid metabolic markers in AD *Drosophila melanogaster* co-treated with probiotics and/or prebiotics with BADGE. (DOCX)

S5 Table Inflammatory markers in AD *Drosophila melanogaster* co-treated with probiotics and/or prebiotics with BADGE. (DOCX)

S1 Fig Survival of APP-BACE1 Alzheimer’s disease *Drosophila* model following Lf5221, probiotic or synbiotic treatment. (TIFF)

S2 Fig Survival *Drosophila* following Lf5221, probiotic or synbiotic treatment. (TIFF)

S3 Fig Metabolic stress following a high-sugar diet stress is alleviated by probiotic and synbiotic formulations. (TIFF)

S4 Fig Metabolic stress following a high-fat diet stress is alleviated by probiotic and synbiotic formulations. (TIFF)

S5 Fig Inflammatory markers are reduced by the synbiotic formulation in AD *Drosophila melanogaster* model. (TIFF)

S6 Fig Survival of APP-BACE1 Alzheimer’s disease *Drosophila* model following treatment with pharmacological inhibition of PPARγ. (TIFF)
