# Supplementary material for: A novel synbiotic delays Alzheimer’s disease onset via combinatorial gut-brain-axis signaling in Drosophila melanogaster
Source: PLoS One. 2019 Apr 22;14(4):e0214985. doi: 10.1371/journal.pone.0214985 (PMC6476497; doi:10.1371/journal.pone.0214985)
Supplement: S1 Table — (DOCX) [file pone.0214985.s001.docx]

**S1 Table:** **Primer sequences to identify various metabolic markers in *Drosophila***

| **Gene Name** | **Sequence** | **Annealing Temp.** |
| --- | --- | --- |
| *Dilp 2* | *F: 3’ –* AGCAAGCCTTTGTCCTTCATCTC – 5’ | 50 °C |
|  | *R: 3’ –* ACACCATACTCAGCACCTCGTTG – 5’ |  |
| *Dilp 3* | *F: 3’ –* TGTGTGTATGGCTTCAACGCAATG – 5’ | 50 °C |
|  | *R: 3’ –* CACTCAACAGTCTTTCCAGCAGGG – 5’ |  |
| *InR* | *F: 5’ –* AACAGTGGCGGATTCGGTT – 3’ | 54 °C |
|  | *R: 5’ –* TACTCGGAGCATTGGAGGCAT – 3’ |  |
| *ACC* | *F: 3’ –* TTAGTCAGCTGCAGGCAAAGG – 5’ | 54 °C |
|  | *R: 3’ –* CGGAAGCTAACGCCACACA – 5’ |  |
| *FAS* | *F: 3’ –* CAACAAGCCGAACCCAGATCTT – 5’ | 50 °C |
|  | *R: 3’ –* CAAAGGAGTTCAGGCCGATGAT – 5’ |  |
| *PEPCK* | *F: 3’ –* CGCCCAGCGACATGGATGCT – 5’ | 60 °C |
|  | *R: 3’ –* GTACATGGTGCGACCCTTCA – 5’ |  |
| *dTOR* | *F: 3’ –* GGCCGTCCAGGTTCAAAAAC - 5’ | 59 °C |
|  | *R: 3’ –* AATCCGGCGATAGTTCCGTC – 5’ |  |
| *dAkt* | *F: 3’ –* GAGTCGTGTGCTCAAGTCCA – 5’ | 59 °C |
|  | *R: 3’ –* TGCATCACAAAACACAGGCG – 5’ |  |
| *dFOXO* | *F: 3’ –* TCGCCGAACTCAGTAACCAC – 5’ | 59 °C |
|  | *R: 3’ –* TCCTATCAAAGTAGAGGCGCA – 5’ |  |
| *SREBP* | F: 5’-GGCAGTTTGTCGCCTGATG-3’ | 56 °C |
|  | R: 5’-CAGACTCCTGTCCAAGAGCTGTT-3’ |  |
| *E75* | F: 5’-CAGTGTCTCTCGTTGCTCA-3’ | 54 °C |
|  | R: 5’-AACCGATTGCTTCGCTCTCT-3’ |  |
| *LSD* | F: 5’-ACTTGTAGTGCCAGTTCCCG-3’ | 52 °C |
|  | R: 5’-ACCAGACTGCTCCACATTCG-3’ |  |
| *Rp49* | *F: 3’ –* AGATCGTGAAGAAGCGCACCAAG – 5 ‘ | 52 °C |
|  | *R: 3’ –* CACCAGGAACTTCTTGAATCCGG – 5’ |  |
