# Supplementary material for: A novel synbiotic delays Alzheimer’s disease onset via combinatorial gut-brain-axis signaling in Drosophila melanogaster
Source: PLoS One. 2019 Apr 22;14(4):e0214985. doi: 10.1371/journal.pone.0214985 (PMC6476497; doi:10.1371/journal.pone.0214985)
Supplement: S2 Table — (DOCX) [file pone.0214985.s002.docx]

**S2 Table: Primer sequences of *Drosophila melanogaster* inflammatory markers**

| **Gene Name** | **Sequence (5’ – 3’)** | **Annealing Temp.** |
| --- | --- | --- |
| *Duox* | *F:* GCTGCACGCCAACCACAAGAGACT | 54 °C |
|  | *R:* CACGCGCAGCAGGATGTAAGGTTT |  |
| *IMD* | *F:* TCGAATGCCAATAATCTGCA | 52 °C |
|  | *R:* CGCGATGCTGGGACTCCCAC |  |
| *Relish* | *F:* TGGGAGGCATACGCAAAGT | 55 °C |
|  | *R:* CAATTACGCTCCGTGGCTTG |  |
| *Attacin A* | *F:* GGCCCATGCCAATTTATTCA | 56 °C |
|  | *R:* CATTGCGCTGGAACTCGAA |  |
| *Diptericin* | *F:* AGGTGTGGACCAGCGACAA | 56 °C |
|  | *R:* TGCTGTCCATATCCTCCATTCA |  |
| *Drosocin* | *F:* GCACAATGAAGTTCACCATCGT | 56 °C |
|  | *R:* CCACACCCATGGCAAAAAC |  |
