# Supplementary material for: A novel synbiotic delays Alzheimer’s disease onset via combinatorial gut-brain-axis signaling in Drosophila melanogaster
Source: PLoS One. 2019 Apr 22;14(4):e0214985. doi: 10.1371/journal.pone.0214985 (PMC6476497; doi:10.1371/journal.pone.0214985)
Supplement: S3 Table — (DOCX) [file pone.0214985.s003.docx]

**S3 Table: Glucose metabolic markers in AD *Drosophila melanogaster* co-treated with probiotics and/or prebiotics with BADGE.** Glucose metabolic markers were assessed in AD *Drosophila melanogaster* co-treated with the probiotic and/or prebiotic formulations and the PPARγ inhibitor BADGE. Total glucose was measured using a colorimetric assay while the mRNA expression of *dilp*2, *dilp*3, InR, dAkt, dTOR and dFOXO was assessed using real-time PCR. Each value is a ratio of change of expression from day 0 to day 30 where each group is the average of n = 5 independent groups +/- geometric mean. Significance is indicated as black stars (*) relative to the control group where * p < 0.05 and ** p < 0.01.

|  | **Control** | **Lf5221** | **TFLA** | **Probiotic** | **Synbiotic** |
| --- | --- | --- | --- | --- | --- |
| **Physiological Marker** | | | | | |
| **Glucose** | 2.19 ± 0.09 | 2.75 ± 0.10* | 2.67 ± 0.16* | 2.33 ± 0.12 | 2.25 ± 0.09 |
| **Genetic Markers** | | | | | |
| ***Dilp 2*** | 1.96 ± 0.21 | 1.36 ± 0.23* | 1.51 ± 0.17* | 0.97 ± 0.23** | 1.54 ± 0.17* |
| ***Dilp 3*** | 3.14 ± 0.19 | 3.53 ± 0.18 | 2.13 ± 0.46** | 3.42 ± 0.19 | 5.33 ± 0.12** |
| ***InR*** | 0.99 ± 0.29 | 1.23 ± 0.28 | 1.11 ± 0.23 | 0.86 ± 0.22 | 1.76 ± 0.23** |
| **dAkt** | 0.18 ± 0.15 | 0.44 ± 0.23 | 0.51 ± 0.20* | 0.64 ± 0.19* | 0.39 ± 0.19 |
| **dTOR** | 2.45 ± 0.18 | 1.46 ± 0.22** | 1.00 ± 0.19** | 1.63 ± 0.21** | 2.02 ± 0.17 |
| **dFOXO** | 1.01 ± 0.17 | 1.01 ± 0.15 | 0.94 ± 0.20 | 1.26 ± 0.27 | 1.71 ± 0.18* |
