# Supplementary material for: A novel synbiotic delays Alzheimer’s disease onset via combinatorial gut-brain-axis signaling in Drosophila melanogaster
Source: PLoS One. 2019 Apr 22;14(4):e0214985. doi: 10.1371/journal.pone.0214985 (PMC6476497; doi:10.1371/journal.pone.0214985)
Supplement: S4 Table — (DOCX) [file pone.0214985.s004.docx]

**S4 Table: Fatty acid metabolic markers in AD *Drosophila melanogaster* co-treated with probiotics and/or prebiotics with BADGE.** Fatty acid metabolic markers were assessed in AD *Drosophila melanogaster* co-treated with the probiotic and/or prebiotic formulations and BADGE. Total triglycerides weremeasured using a colorimetric assay while the expression of *ACC, FAS, PEPCK, Lsd2, SREBP* and *E75* was assessed using real-time PCR. Each value is a ratio of change of expression from day 0 to day 30 where each group is the average of n = 5 independent groups +/- geometric mean. Significance is indicated as black stars (*) relative to the control group where * p < 0.05 and ** p < 0.01.

|  | **Control** | **Lf5221** | **TFLA** | **Probiotic** | **Synbiotic** |
| --- | --- | --- | --- | --- | --- |
| **Physiological Markers** | | | | | |
| **Trigly.** | 1.82 ± 0.11 | 1.34 ± 0.08* | 1.42 ± 0.15* | 1.46 ± 0.12* | 1.79 ± 0.07 |
| **Genetic Markers** | | | | | |
| ***ACC*** | 1.58 ± 0.12 | 0.70 ± 0.15** | 0.82 ± 0.22** | 0.98 ± 0.23* | 0.98 ± 0.20* |
| ***FAS*** | 1.52 ± 0.14 | 1.79 ± 0.15 | 1.56 ± 0.21 | 2.01 ± 0.15* | 1.80 ± 0.24 |
| ***PEPCK*** | 0.77 ± 0.25 | 0.82 ± 0.25 | 0.62 ± 0.24 | 0.97 ± 0.26 | 0.54 ± 0.26 |
| **Lsd2** | 0.43 ± 0.18 | 0.47 ± 0.13 | 0.31 ± 0.08 | 0.45 ± 0.07 | 0.37 ± 0.09 |
| **SREBP** | 0.67 ± 0.03 | 0.71 ± 0.04 | 0.49 ± 0.02 | 0.60 ± 0.03 | 0.81 ± 0.03 |
| **E75** | 0.31 ± 0.02 | 0.17 ± 0.02* | 0.17 ± 0.02* | 0.23 ± 0.04 | 0.60 ± 0.10* |
