# Supplementary material for: A novel synbiotic delays Alzheimer’s disease onset via combinatorial gut-brain-axis signaling in Drosophila melanogaster
Source: PLoS One. 2019 Apr 22;14(4):e0214985. doi: 10.1371/journal.pone.0214985 (PMC6476497; doi:10.1371/journal.pone.0214985)
Supplement: S5 Table — (DOCX) [file pone.0214985.s005.docx]

**S5 Table: Inflammatory markers in AD *Drosophila melanogaster* co-treated with probiotics and/or prebiotics with BADGE.** Inflammatory markers were assessed in AD *Drosophila melanogaster* co-treated with the probiotic and/or prebiotic formulations and BADGE. The agar diffusion test was used to test the production of antimicrobial factors from *Drosophila* in plates inoculated with both *S. aureus* or *E. coli*. Further, the gene expression of a variety of inflammatory markers was assessed by real-time PCR including *duox, IMD, Relish, Attacin A, Defensin* and *Diptercin*. Each value is a ratio of change of expression from day 0 to day 30 where each group is the average of n = 5 independent groups +/- geometric mean. Significance is indicated as black stars (*) relative to the control group where * p < 0.05 and ** p < 0.01.

|  | **Control** | **Lf5221** | **TFLA** | **Probiotic** | **Synbiotic** |
| --- | --- | --- | --- | --- | --- |
| **Agar Diffusion Test** | | | | | |
| ***S. aureus*** | 0.63 ± 0.09 | 0.71 ± 0.10 | 0.67 ± 0.08 | 0.79 ± 0.09 | 0.80 ± 0.07 |
| ***E. coli*** | 0.69 ± 0.07 | 0.73 ± 0.07 | 0.77 ± 0.07 | 0.80 ± 0.10 | 0.82 ± 0.08 |
| **Genetic Markers** | | | | | |
| ***Duox*** | 0.36 ± 0.02 | 0.46 ± 0.03 | 0.44 ± 0.02 | 0.37 ± 0.02 | 0.11 ± 0.02* |
| ***IMD*** | 0.73 ± 0.14 | 0.96 ± 0.23 | 0.87 ± 0.23 | 0.59 ± 0.21 | 0.62 ± 0.18 |
| ***Relish*** | 1.02 ± 0.11 | 1.51 ± 0.15* | 1.34 ± 0.14 | 1.40 ± 0.16* | 1.69 ± 0.11* |
| **Attacin A** | 3.98 ± 0.43 | 5.53 ± 0.51* | 4.58 ± 0.45* | 2.52 ± 0.45* | 3.03 ± 0.44 |
| **Defensin** | 1.77 ± 0.41 | 1.20 ± 0.37 | 2.31 ± 0.20* | 2.08 ± 0.17 | 1.46 ± 0.12 |
| **Diptercin** | 4.35 ± 0.35 | 5.88 ± 0.35 | 4.21 ± 0.05 | 4.59 ± 0.05 | 4.30 ± 0.13 |
