# Supplementary figures and images for: A novel synbiotic delays Alzheimer’s disease onset via combinatorial gut-brain-axis signaling in Drosophila melanogaster
Source: PLoS One. 2019 Apr 22;14(4):e0214985. doi: 10.1371/journal.pone.0214985 (PMC6476497; doi:10.1371/journal.pone.0214985)

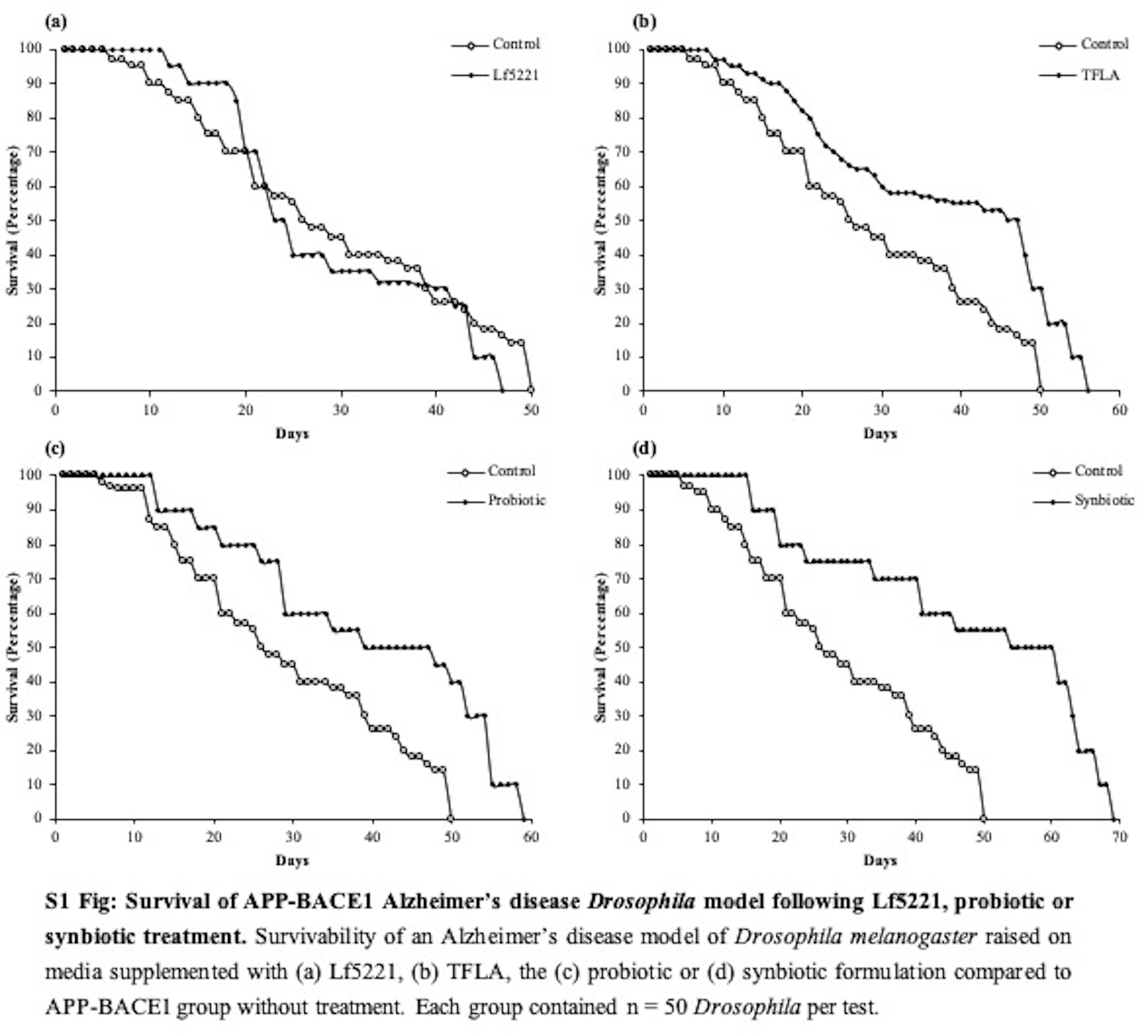

Supplement: S1 Fig — (TIFF) [file pone.0214985.s006.tiff]

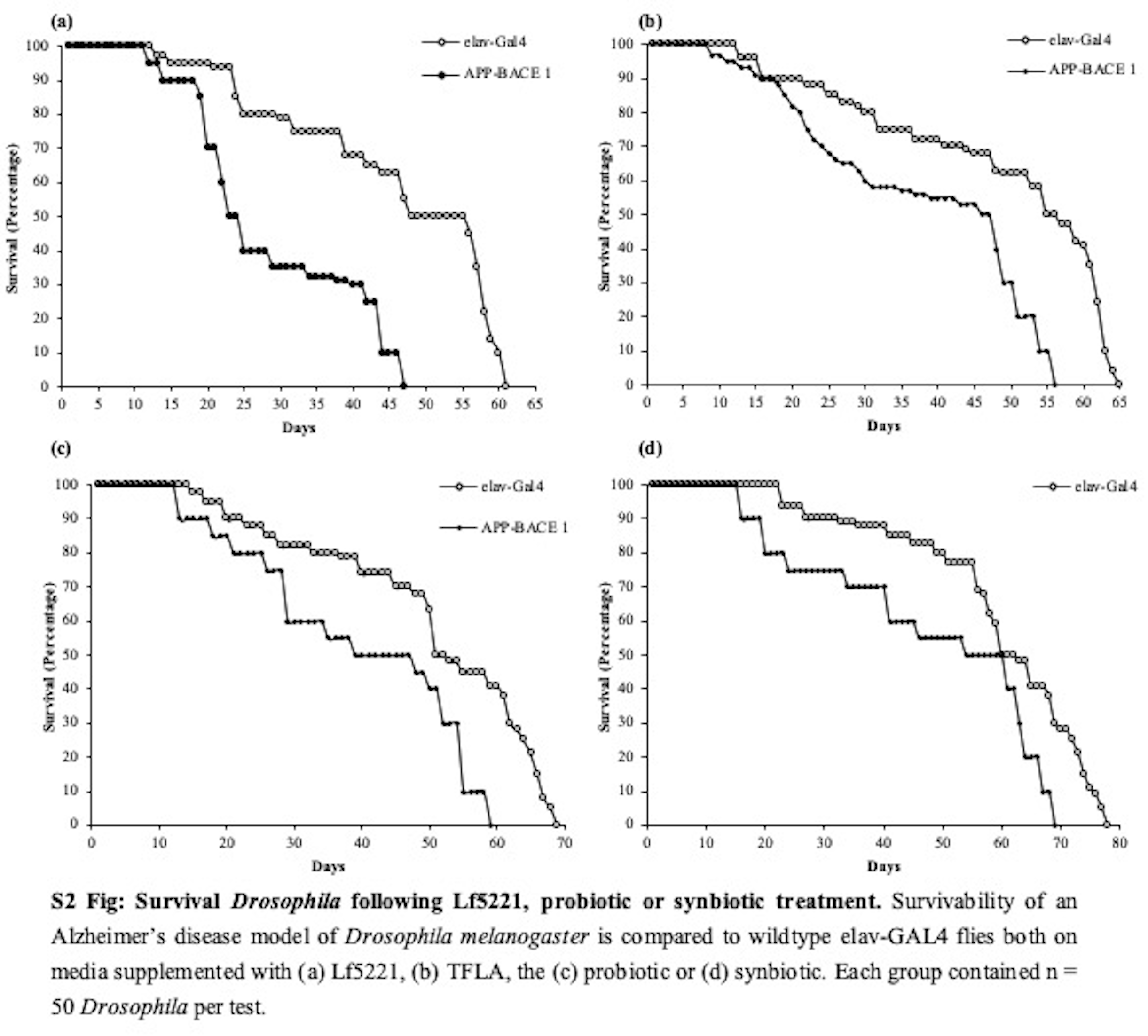

Supplement: S2 Fig — (TIFF) [file pone.0214985.s007.tiff]

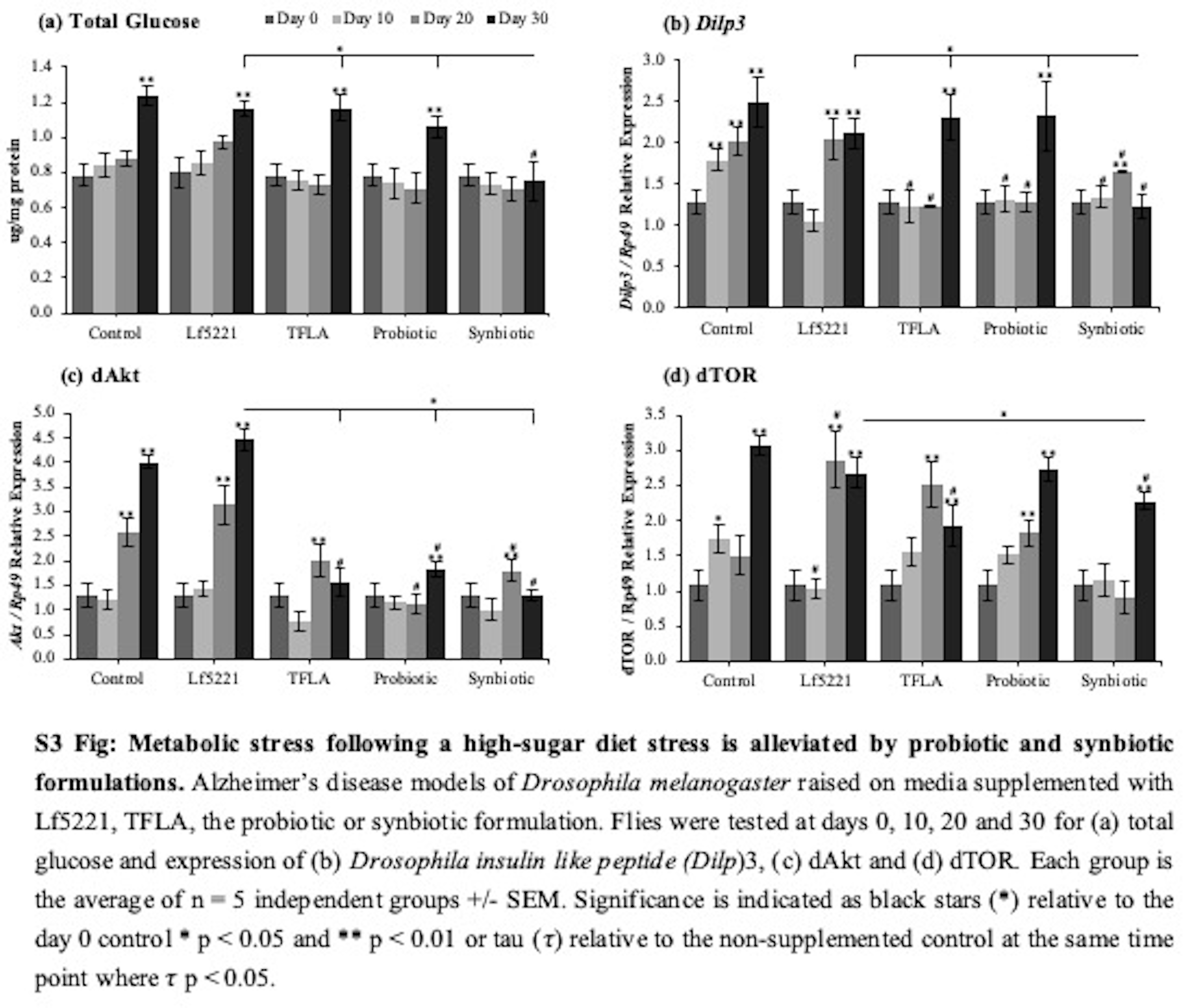

Supplement: S3 Fig — (TIFF) [file pone.0214985.s008.tiff]

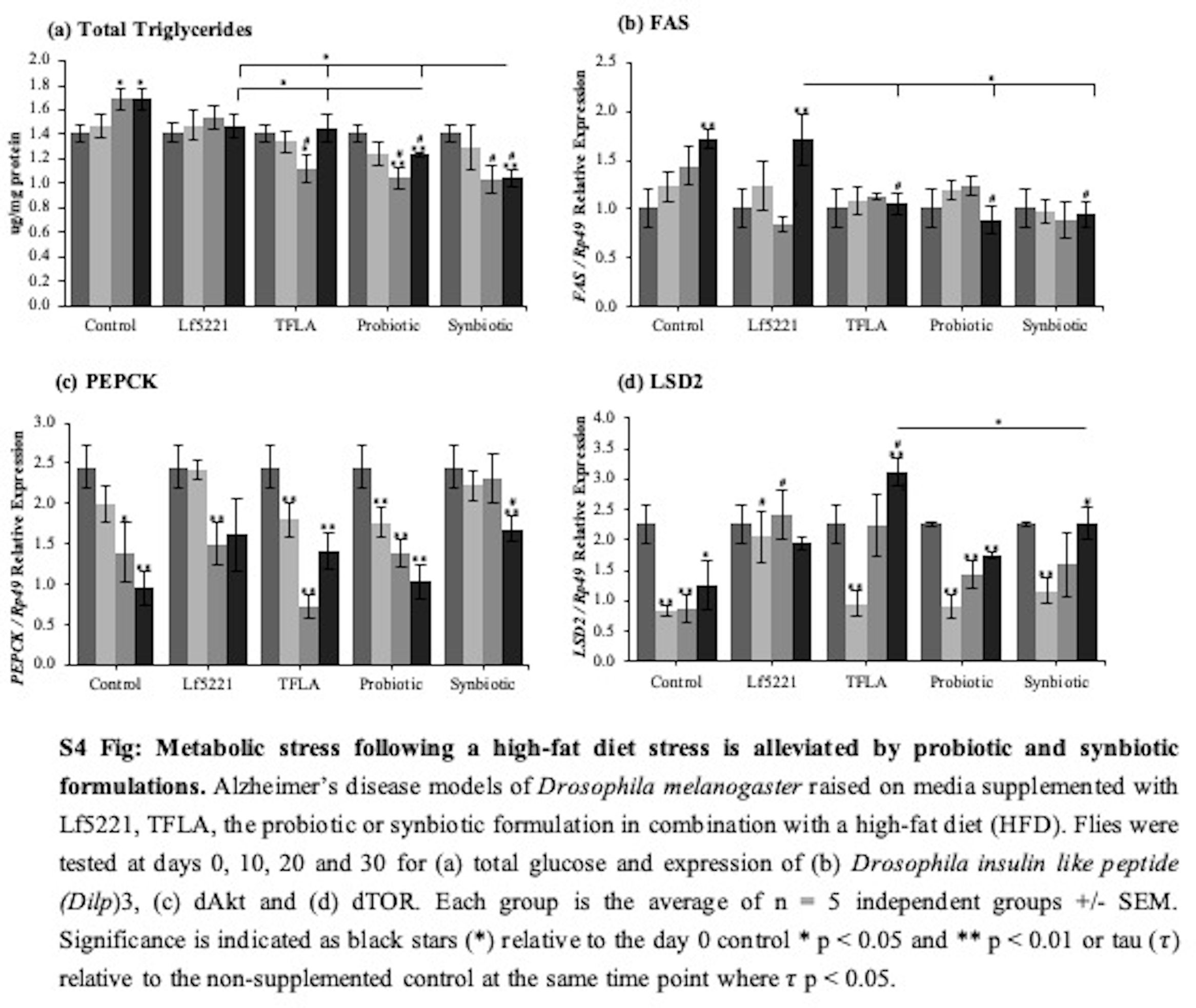

Supplement: S4 Fig — (TIFF) [file pone.0214985.s009.tiff]

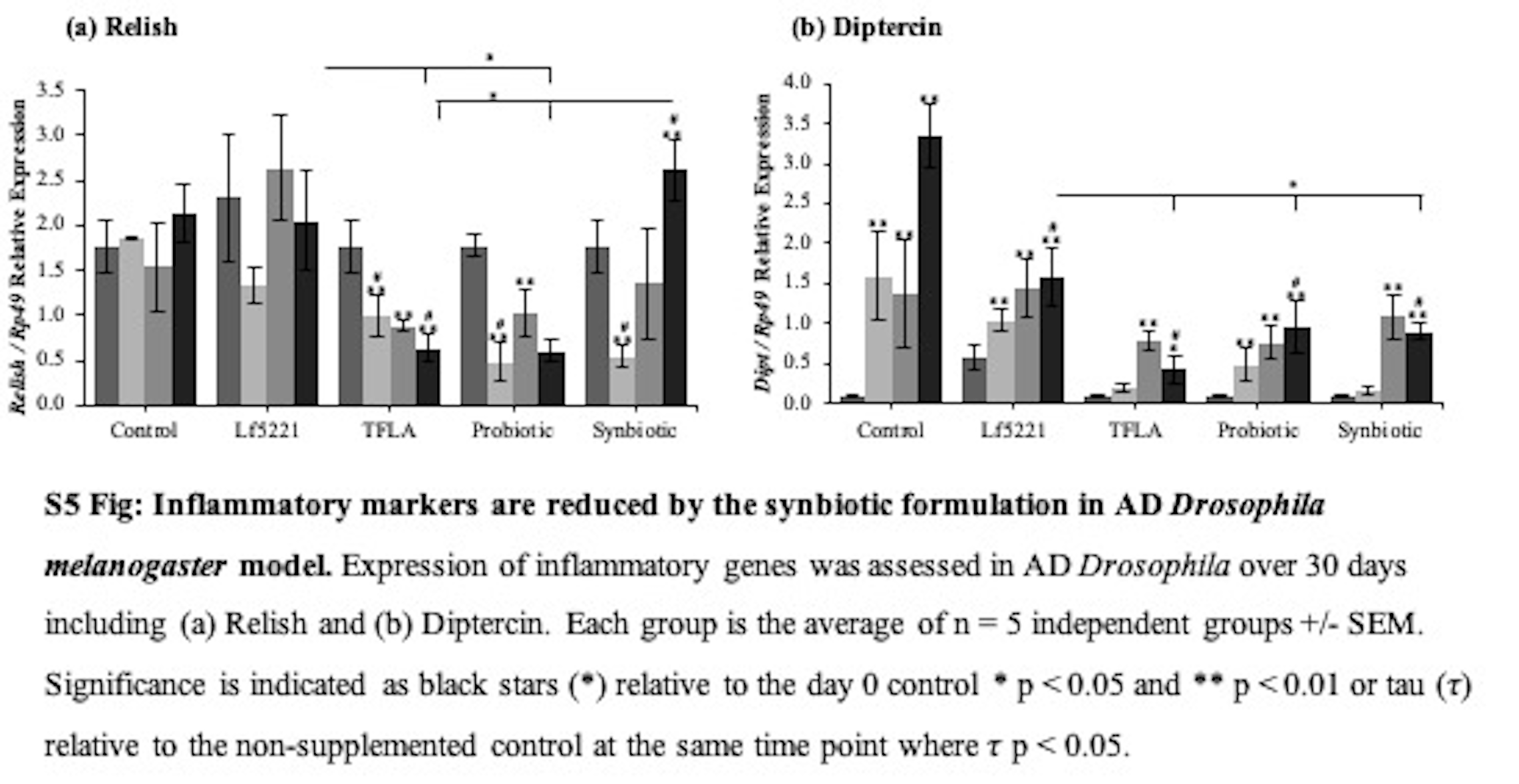

Supplement: S5 Fig — (TIFF) [file pone.0214985.s010.tiff]

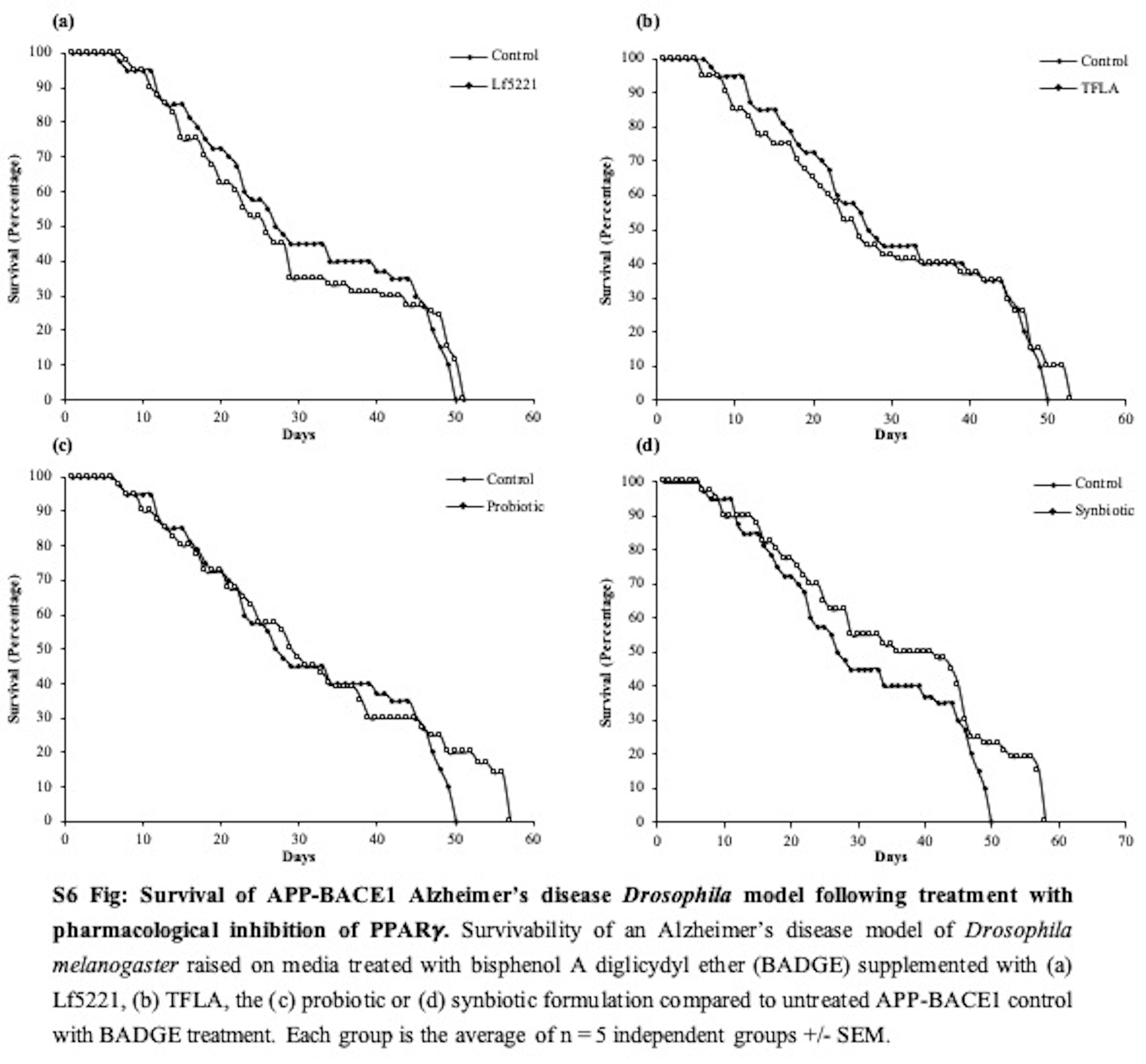

Supplement: S6 Fig — (TIFF) [file pone.0214985.s011.tiff]
